# Nanocellulose-Bovine Serum Albumin Interactions in an Aqueous Medium: Investigations Using In Situ Nanocolloidal Probe Microscopy and Reactive Molecular Dynamics Simulations

**DOI:** 10.1021/acs.biomac.4c00264

**Published:** 2024-05-28

**Authors:** Houssine Khalili, Susanna Monti, Edouard Pesquet, Aleksander Jaworski, Salvatore Lombardo, Aji P Mathew

**Affiliations:** † Department of Materials and Environmental Chemistry, Stockholm University, Stockholm 10691, Sweden; ‡ CNR-ICCOM, Institute of Chemistry of Organometallic Compounds, via Moruzzi 1, Pisa 56124, Italy; § Department of Ecology, Environment and Plant Sciences, Stockholm University, Stockholm 10691, Sweden

## Abstract

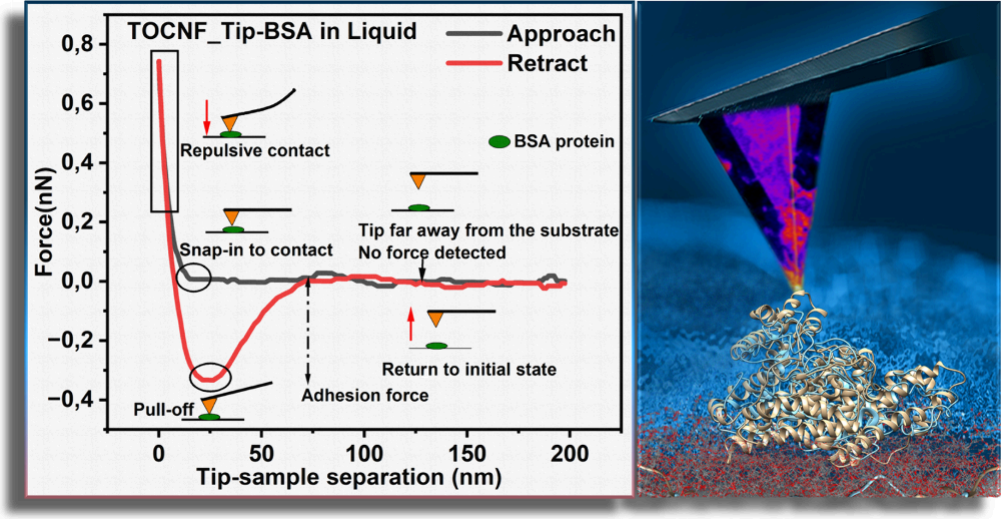

As a versatile nanomaterial derived from renewable sources, nanocellulose has attracted considerable attention for its potential applications in various sectors, especially those focused on water treatment and remediation. Here, we have combined atomic force microscopy (AFM) and reactive molecular dynamics (RMD) simulations to characterize the interactions between cellulose nanofibers modified with carboxylate or phosphate groups and the protein foulant model bovine serum albumin (BSA) at pH 3.92, which is close to the isoelectric point of BSA. Colloidal probes were prepared by modification of the AFM probes with the nanofibers, and the nanofiber coating on the AFM tip was for the first time confirmed through fluorescence labeling and confocal optical sectioning. We have found that the wet-state normalized adhesion force is approximately 17.87 ± 8.58 pN/nm for the carboxylated cellulose nanofibers (TOCNF) and about 11.70 ± 2.97 pN/nm for the phosphorylated ones (PCNF) at the studied pH. Moreover, the adsorbed protein partially unfolded at the cellulose interface due to the secondary structure’s loss of intramolecular hydrogen bonds. We demonstrate that nanocellulose colloidal probes can be used as a sensitive tool to reveal interactions with BSA at nano and molecular scales and under in situ conditions. RMD simulations helped to gain a molecular- and atomistic-level understanding of the differences between these findings. In the case of PCNF, partially solvated metal ions, preferentially bound to the phosphates, reduced the direct protein–cellulose connections. This understanding can lead to significant advancements in the development of cellulose-based antifouling surfaces and provide crucial insights for expanding the pH range of use and suggesting appropriate recalibrations.

## Introduction

Nanocellulose has unique surface chemistry, nanostructured morphology, and excellent versatility as a biobased functional material for various environmental applications. For example, water treatment is a critical field where nanocellulose can be propitious.^1,2^ Its potential is primarily achieved through surface chemical modification,^3^ which gives it tunable properties by introducing specifically active functional groups. Nanocellulose engineered with negatively charged surface groups, such as carboxylates and phosphates, can efficiently capture positively charged water pollutants such as dyes^4,5^ and various heavy metals.^6−9^

In this context, the hydrophilic TEMPO (2,2,6,6-tetramethylpiperidine-1-oxylradical)-mediated oxidation nanofibers (TOCNFs), with a high aspect ratio, a specific surface area exceeding 600 m^2^/g,^10^ and abundance of negative surface charges (carboxyl groups, 1.7 mmol/g), is extensively employed for water treatment. The alternative cellulose functionalization with phosphate groups (ROPO_3_^2–^), obtained through esterification^11^ using phosphorylating agents such as phosphoric acid (H_3_PO_4_) and diammonium hydrogen phosphate ((NH_4_)_2_HPO_4_), is also effective for capturing water effluents.^12,13^

The interactions at nanocellulose surfaces with a wide range of pollutants have been examined in several works and rationalized from a thermodynamical perspective in a recent review. It revealed an entropy-driven mechanism associated with releasing surface-structured water molecules from the electronic double layer formed upon binding.^14^ The advantages of carboxylated and phosphated nanocelluloses are their pH-dependent interactions with positively charged pollutants, which can be directly regenerated.^15,16^

Since BSA does not bind to cellulose,^17^ there is a growing interest in understanding this system. Some works try to induce this interaction by a modification process to make the binding process favorable.^18^ There are only a few studies on BSA interaction with nanocellulose; most of these studies were conducted at neutral pH, where BSA has a negative surface charge due to its isoelectric point (pHi) of 4.7.^19^ Aguilar-Sanchez et al. employed the quartz crystal microbalance with dissipation monitoring (QCM-D) to study the attachment of BSA to nanocellulose, directly assessing this phenomenon and highlighting the significant role of nanocellulose’s natural affinity for water in reducing this interaction.^20−22^ Valencia et al. introduced an in situ technique that involved monitoring of changes in pore size in operando using synchrotron radiation-based SAXS. They found that when the nonmodified membranes were employed to filter the feed containing BSA, the pore radius decreased compared to the zwitterionic-modified membrane.^23^

AFM-based force spectroscopy can directly quantify the interaction forces and provide direct information on the interaction with the foulants in wet conditions. Additionally, it can serve as a versatile instrument for examining surface characteristics, such as roughness,^22,24^ distribution of pore sizes,^25^ nanomechanical properties, etc.^26^ Recently, Eskhan et al.^27^ have used the AFM colloidal probe technique combined with QCM-D to examine the biofouling process of two distinct biomolecular species, namely, BSA (MW = 66.43 kDa, pHi = 4.7) and humic acid (HA), in contact with different commercial membranes. The results indicated a higher adhesion strength between BSA and the membranes. In an earlier investigation by Zhu et al., they successfully functionalized AFM probes with oxidized cellulose nanofibers and cellulose nanocrystals (CNC) mediated by TEMPO to examine the interaction with Cu(II) ions and the Victoria blue B dye (MW = 506 Da) in a liquid environment.^28^

Herein, we report on two new types of nanocellulose-modified AFM probes with carboxylic and phosphate functionalities to determine the intermolecular interaction with BSA at a low pH where BSA has a positive surface charge. The study provides precious indications of the potential use of nanocellulose for generating low-fouling surfaces below the isoelectric point. We examined these interactions' in situ conditions, both in a liquid phase and air. We used the peak force quantitative nanomechanics (PFQNM) mode for force–distance curves and nanomechanical maps to fast-track this exploration. The significance of this work is in developing sophisticated methods for functionalizing and characterizing nanocellulose-modified AFM probes. These probes are then used to showcase their interactions with BSA, and this approach can be expanded to investigate surface interactions in real-time conditions with various materials. In this case, we resorted to atomistic computational techniques to help the experimental interpretation of BSA-functionalized nanocellulose interactions. More specifically, reactive molecular dynamics simulations (RMD) based on efficiently parametrized force fields enabled the description of inorganic and organic components in dry and wet conditions, their reactions, and the variation of protonation states of the functionalizing cellulose chains in response to the local environment. We started from our earlier cellulose fiber supramolecular models used for disclosing the structure and dynamics of nanocellulose-graphene oxide sheet complexes and their ability to capture metal ions^29^ and further extended the representations to hybrid matrices with various functionalizations, including methylated, carboxylated, and phosphorylated chains.^30^ The matrices were employed to investigate the possible antifouling properties of cellulose fibers covered with fatty acids, evidencing their tendency to mitigate cellulose hydrophilicity based on the concentration. Indeed, the carboxyl/phosphate-functionalized fibers have a hydrophilic character that could attract polar molecules and positively charged metal ions. Thus, adding a hydrophobic partial cover determined the appearance of large hydrophobic regions on cellulose interfaces that could mitigate the attraction and even reduce fouling.^31^

For this study, we prepared new configurations of the phosphorylated and carboxylated cellulose fibers (hereafter named PCNF and TOCNF) corresponding to the experimental data. We followed the procedure already used in a previous study^31^ to simulate possible adsorption mechanisms of BSA on the cellulose interfaces. Explicit water molecules and sodium counterions rendered the moisture environment at the experimental pH at the cellulose–protein interface. Then, we performed the RMD based on a preparametrized force field tuned for cellulose and protein systems,^31^ to explore the adsorption dynamics of the BSA onto the moist cellulose surface, its binding mode, and the evolution of the protonation state of the amino acids at the interface.

## Materials and Methods

### Experimental Section: Materials

TEMPO-oxidized cellulose nanofibers (TOCNF, carboxyl content of 1 mmol/g) and phosphorylated cellulose nanofibers (PCNF, 3 mmol/g) were prepared from softwood cellulose fibers (Norwegian spruce) with a high cellulose content (95% cellulose, 4.5% hemicellulose-type galactoglucomannan, and 0.1% lignin content as provided by Domsjö Fabriker AB, Sweden) following the procedures described by Isogai et al.^32^ and Hadid et al.,^33^ respectively. Other chemicals chloroform, phosphate-buffered saline (PBS) solution (pH ∼7.4), (3-aminopropyl)triethoxysilane (APTES), triethylamine (TEA), bovine serum albumin protein (BSA) lyophilized powder >96% with a molecular weight (MW) of 66.43 kDa (40 × 140 Å), glutaraldehyde (GA) solution, 50 wt % in H_2_O, diammonium phosphate (DAP), urea (CO(NH_2_)_2_), sodium hydroxide (NaOH), hydrochloric acid (HCl, 37%), TEMPO, sodium bromide, and a 2 M sodium hypochlorite solution and the reagents used for the fluorescence labeling with rhodamine B isothiocyanate including DMSO, pyridine 99%, ethanol, and dibutyltin dilaurate were all purchased from Sigma-Aldrich.

### AFM Probe and Substrate Functionalization

The AFM probes, which are made of silicon nitride (Si_3_N_4_), naturally undergo oxidation under ambient conditions and develop a layer of silicon dioxide with silanol groups (Si–OH).^34^ To generate more hydroxyl groups necessary for subsequent amino modification through silanization with APTES in a gas phase and to remove any susceptible contaminants, the probes underwent a cleaning process. This was performed using a UV-ozone chamber followed by immersion in chloroform for 5 min. Afterward, the probes were placed in a desiccator with two separate Eppendorf tubes under an inert atmosphere. One tube contained 30 μL of APTES, while the other contained 10 μL of TEA. They were left in this setup for 2 h. Subsequently, the amino-modified probes were removed from the desiccator, and nanocellulose suspensions (0.4 wt % PCNF and TOCNF) were applied to the probes and kept in contact for 1 h. Afterward, a washing process with water was conducted to remove the unbound excess. This resulted in PCNF_tip and TOCNF_tip, denoting the respective modifications.

**Figure 1:**
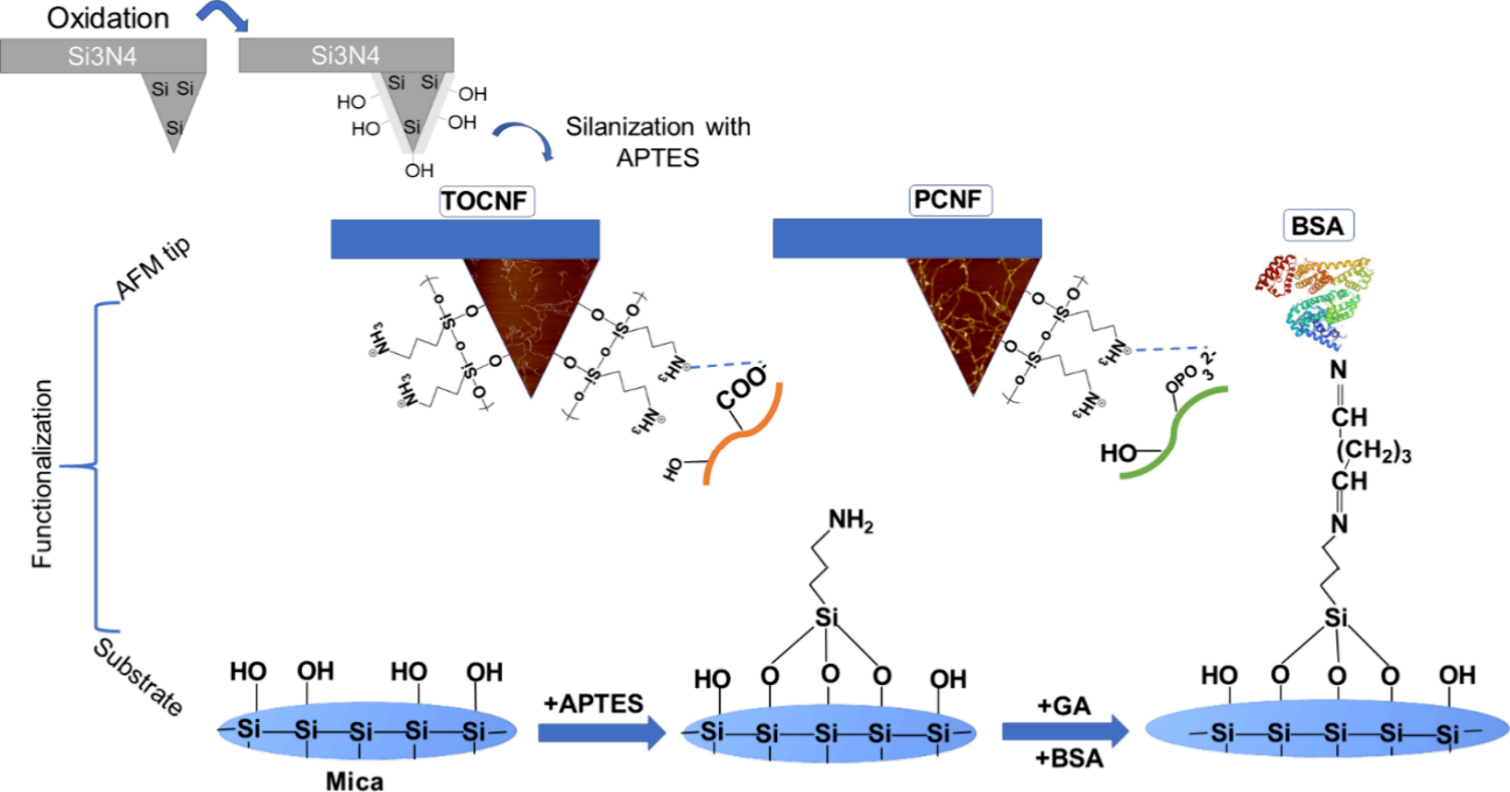
Schematic illustration of the functionalization process of both AFM probes and the mica substrate developed in this work.

On the other hand, small aliquots of glutaraldehyde solution (0.001M) were applied to a freshly cleaved and APTES-coated mica substrate to prepare the BSA-modified mica substrate. This was followed by adding a BSA solution (5 mg/L at pH = 3.92 of PS) and then washing it three times to remove unbonded protein. The full procedure is summarized in Figure 1.

### Fluorescence Labeling

TOCNFs were grafted with rhodamine B isothiocyanate using a previously described procedure.^35^ TOCNFs (1 g) were dispersed in 40 mL of DMSO by stirring at 65 °C. Rhodamine B isothiocyanate (7.5 mg) was added. Afterward, 50 μL of dibutyltin dilaurate was added after a few drops of pyridine. The reaction was left to stir at 65 °C for 2 h in the dark. Finally, the product was washed with ethanol dialyzed in water and then stored in a dark condition. Before confocal microscopy imaging, the fluorescent suspension was applied to the AFM probes.

### AFM Force Measurements

The force measurements were conducted using a Bruker Dimension FastScan atomic force microscopy (AFM) instrument, equipped with a NanoScope controller, Santa Barbara, California, USA. The measurements were performed in both air and liquid environments, utilizing the peak force quantitative nanomechanics (PFQNM) mode. Prior to the measurements, the AFM probes were calibrated using the thermal tune method to determine their deflection sensitivity and spring constant. The tip radius was also determined using high-resolution scanning electron microscopy (SEM). To ensure the reliability of the results, all experiments were repeated three times for reproducibility. The setup parameters for all the experiments were consistent and set as follows: the scan size was 1 μm, the scan rate was 0.2 Hz, the tip velocity 0.2 μm/s ramp size was 200 nm, the trigger threshold was 0.1 V, the number of samples was 512, and the *Z* closed loop was enabled. Consequently, a collection of force curves was acquired and analyzed.

### Characterization Techniques

#### Atomic Force Microscopy (AFM)

The cellulose nanofibers’ morphology was visualized using a Dimension FastScan AFM instrument, equipped with a NanoScope controller manufactured by Bruker in Santa Barbara, California, USA. The TESPA-V2 probe, which had a spring constant of *k* = 42 N/m and a nominal tip radius of 7 nm, was utilized in the dynamic mode, specifically the peak force tapping mode. The suspensions were diluted to a concentration of 0.001 wt % and subjected to 15 min of sonication to prepare the sample for AFM analysis. Subsequently, the diluted suspensions were spin-coated onto a freshly cleaved and APTES-coated mica substrate, which was affixed to the AFM metal disc using double-sided tape. ScanAsyst Air probes with a spring constant of 0.4 N/m and a nominal tip radius of 2 nm were employed for functionalization and force measurement in air. In liquid, SNL-C probes with a spring constant of 0.24 N/m and a nominal tip radius of 2 nm were used. The acquired data were processed using Nanoscope Analysis 1.5 software.

#### Scanning Electron Microscopy-Energy-Dispersive X-ray Spectroscopy (SEM-EDS)

To examine the condition of the AFM probes before and after functionalization and to measure their tip radius, scanning electron microscopy (SEM) was employed, using a Jeol JSM-7000 F microscope from Japan, operating at an accelerating voltage of 5 kV. The AFM probes were observed without any coating. In addition, energy-dispersive X-ray spectroscopy (EDS) was utilized to provide elemental analysis of the probes.

#### Fluorescence Microscopy

Fluorescence confocal imaging was performed using a Zeiss LSM800 equipped with a laser diode array. AFM probes were imaged using long-distance 10× air objectives to perform either *xyz* optical sectioning or *xy*λ fluorescence emission spectra measurements.

### Characterization of Nanocellulose Materials

#### Conductometric Titration

Around 0.3 g of PCNF and TOCNF was diluted in distilled water. The mixture was then homogenized using an ultrasonic device. Additionally, the pH of the solution was adjusted by adding 6 mL of HCl (0.1M), after which a conductometric titration was carried out using NaOH with a conductivity meter (SevenExcellence, Mettler Toledo).

#### Zeta Potential

The zeta potentials of TOCNF and PCNF nanocellulose suspensions at a concentration of 0.001 wt %, as well as BSA solution at a concentration of 5 mg/L and a range of pH, were measured in water and PS solution using a Zetasizer Nano ZS instrument manufactured by Malvern in the United Kingdom. The measurements were conducted at a temperature of 25 °C.

#### Nuclear Magnetic Resonance (NMR) Spectroscopy

Magic-angle spinning (MAS) NMR experiments were performed on a Bruker Avance-III spectrometer using 4 mm probe heads and a 14 kHz MAS rate. The ^1^H–^13^C cross-polarization (CP) experiments were performed at a magnetic field of 14.1 T (Larmor frequencies of 600.1 and 150.9 MHz for ^1^H and ^13^C, respectively) and involved Hartmann–Hahn matched ^1^H and ^13^C radio frequency fields applied for a 1.5 ms contact interval for the cross-polarization step and 63 kHz SPINAL-64 proton decoupling. Signal transients (16,384) with a 2 s relaxation delay were collected for each sample. ^13^C Chemical shifts were referenced to tetramethylsilane (TMS). The ^31^P MAS spectra were collected at a magnetic field of 9.4 T (Larmor frequency of 161.9 MHz) using a 90-degree excitation pulse of 2.5 μs, and 64 scans were collected with a relaxation delay of 60 s. ^31^P Chemical shifts were referenced with respect to phosphoric acid (H_3_PO_4_). To interpret the ^31^P signals, three hypothetical models of cellulose (poly)phosphates were evaluated. Calculations were performed with the ORCA code.^36,37^ Geometry optimizations of the models and the subsequent (GIAO) NMR shift calculations were performed at the revPBE-D4/pcseg-1 and PBE0/pcSseg-2 levels of theory, respectively.^38^

#### Fourier Transform Infrared (FTIR) Spectroscopy

To verify the existence of functional groups in both types of nanocelluloses, Fourier transform infrared (FTIR) spectroscopy was employed. The measurements were conducted using a Varian 670-IR spectrometer equipped with an attenuated total reflectance (ATR) accessory. Each spectrum was recorded within the range of 4000 to 500 cm^–1^, with a resolution of 4 cm^–1^ and an accumulation of 32 scans.

#### X-ray Diffraction (XRD)

The semicrystalline structure and crystallinity of the cellulosic materials were examined using an X-ray diffractometer apparatus, specifically the D8 Discover powder diffractometer manufactured by Bruker. The instrument was operated at a voltage of 40 kV and a current of 40 mA, employing monochromatic Cu Kα radiation with a wavelength of 0.154 nm. The cellulosic materials’ crystallinity index (CrI) was determined using Segal’s equation.^39^

#### Thermogravimetric Analysis (TGA)

The cellulosic samples were heated up from 25 to 600 °C under a nitrogen atmosphere at a flow rate of 30 mL/min with a heating rate of 10 °C/min using Discovery TGA (TA Instruments).

### Computational Chemistry

#### Model Building: Cellulose Supports

Models of the cellulose fibers randomly functionalized with carboxyl and phosphate groups (at the experimental concentration) were built using an optimized geometry that consisted of aligned glucosyl chains organized in a rod-like configuration.^24,28,29,40^ Four fibers were packed in a parallel orientation to create realistic portions of the cellulose material large enough to host the BSA protein. After geometry optimizations, to adjust the local arrangements of the functional chains, we performed a series of molecular dynamics simulations in the NPT ensemble at ambient temperature and different pressures to get stable packed fibers (final box size: approximately 85 × 89 × 26 Å^3^). Then, we extended the box side in the *z* direction to 600 Å, and we added two layers of water molecules (thickness ≈ 5 Å) and counterions that neutralized the negative charge of the interface. These configurations were relaxed to remove the bulk organization due to the periodic replicas and get a more reasonable orientation of the carboxyl/phosphate chains (NVT ensemble at *T* = 300 K). We obtained two different model interfaces that could be used for protein adsorption. The side chains of all the glucosyl units at both interfaces rearranged their relative orientations and extended toward the solvent.

#### BSA Model

A plausible model of the BSA protein at the experimental pH of 3.92 was created starting from the 3v03 BSA crystal structure (https://www.rcsb.org)^41^ and resorting to an automated protocol available on the web [http://biophysics.cs.vt.edu/H++] to add the missing hydrogen atoms at the selected pH.^42−44^ The resulting structure had a total charge of +16, which was neutralized in the final model, including the cellulose interface, by balancing the number of Na^+^ counterions.

Before starting the adsorption simulations, we examined the behavior of the protein in a water solution at pH 7. In this case, the total charge was −9. A comparison of the protonation state of the various amino acids at the two different pHs is reported in Table S1 of the Supporting Information. There, it is apparent that the most significant variations involve glutamic acid (at pH 3.92, 24% of GLU becomes neutral) and histidines (at pH 3.92, all become protonated).

To prepare the BSA structure for the adsorption simulations, we inserted the model at pH 7 in a TIP3P water box (112 × 81 × 94 Å^3^) containing 9 Na^+^ counterions and approximately 20,000 water molecules and energy-minimized it. Then, we simulated the whole system in the NPT ensemble at 300 K for around 1 ns and then in the NVT ensemble for another nanosecond using the ff14SB force field and Amber16 software [D. A. Case, R.M. Betz, D.S. Cerutti, T.E. Cheatham, III, T.A. Darden, R.E. Duke, T.J. Giese, H. Gohlke, A.W. Goetz, N. Homeyer, S. Izadi, P. Janowski, J. Kaus, A. Kovalenko, T.S. Lee, S. LeGrand, P. Li, C. Lin, T. Luchko, R. Luo, B. Madej, D. Mermelstein, K.M. Merz, G. Monard, H. Nguyen, H.T. Nguyen, I. Omelyan, A. Onufriev, D.R. Roe, A. Roitberg, C. Sagui, C.L. Simmerling, W.M. Botello-Smith, J. Swails, R.C. Walker, J. Wang, R.M. Wolf, X. Wu, L. Xiao, and P.A. Kollman (2016), AMBER 2016, University of California, San Francisco]. The protein was stable in solution and did not deviate much from the original configuration, having a root-mean-square deviation of the Cα (trace) of approximately 1.2 Å. It preserved its secondary structure (helices) and all the disulfide bridges (Figure S4 of the Supporting Information).

An average geometry, calculated from the last 50 ps of the NVT trajectory in water solution, was used to investigate BSA adsorption modes in moisture conditions on the cellulose models. The BSA protonation state was appropriately revised for the acidic pH (Table S2), and the molecule was deposited close to the moist cellulose surface (covered with water and Na^+^ counterions that neutralized the charged head groups) at a 6 Å surface separation distance in various orientations, generating different initial system configurations. The simulation box (87 × 114 × 600 Å^3^ size) contained approximately 340 Na^+^ counterions and 2000 water molecules. These geometries were energy-minimized and simulated enough in the NVT ensemble at 300 K to get equilibrated complexes where BSA was adsorbed on the cellulose interface (the total simulation time was at most 1 ns). After discarding the configurations where BSA remained far from the interface, we reduced the models to seven representative complexes for each cellulose type (TOCNF and PCNF). The 14 final structures are shown in Figure S6 of the Supporting Information.

#### Reactive Molecular Dynamics Simulation Details

The assembled configurations were equilibrated at ambient temperature for hundreds of picoseconds and sampled in the NVT ensemble for at most 1 ns. No restraints were applied to the systems, and reactivity was always on to simulate bond breaking and formation in response to the surrounding environment. All the MD simulations were performed with Amsterdam Density Functional (ADF)/ReaxFF software [ADF/ReaxFF 2019.3, SCM, Theoretical Chemistry, Vrije Universiteit, Amsterdam, The Netherlands, http://www.scm.com (ReaxFF release adf2019.102)]. The reactive force field parameters to describe cellulose were those already employed in earlier studies,^9,23,28,29,40^ and the protein parameters were extracted from the CHONSMgPNaCuCl_v2 force field,^45^ available in the ADF/ReaxFF package. System configurations were collected every 0.1 ps. Temperature and pressure were regulated through Berendsen’s thermostat and barostat with relaxation constants of 0.5 ps, and the time step was set to 0.25 fs.

#### Analysis of the Sampled Data

The trajectory analysis was carried out on the final stable structures and mainly focused on characterizing the protein adsorption on the cellulose surface. To evaluate the conformational changes of BSA upon adsorption, we calculated the root-mean-square deviation of the atoms of the trace (C_α_) after superimposing the original and adsorbed models (Figure S5), which shows this superimposition in the best interacting model. We single out the residues at the protein’s surface by calculating the solvent-accessible surface areas and estimated the number and types of those residues in contact with the cellulose interface (all the atoms within 5.5 of the cellulose surface). The interaction energy of the adsorbed portion of the protein with the solvated cellulose surfaces was estimated by an energy difference: the energy of the complex minus the sum of the energies of the isolated BSA and cellulose models. These differences were converted to interaction energy per atom to compare the binding strength of the various systems.

Visual examination of the complexes was fundamental to characterize contact regions, adsorption trends, and the behavior of the water molecules and counterions at the interface.

## Results and Discussion

### Characteristics of Functionalized Cellulose Nanofibers

**Figure 2:**
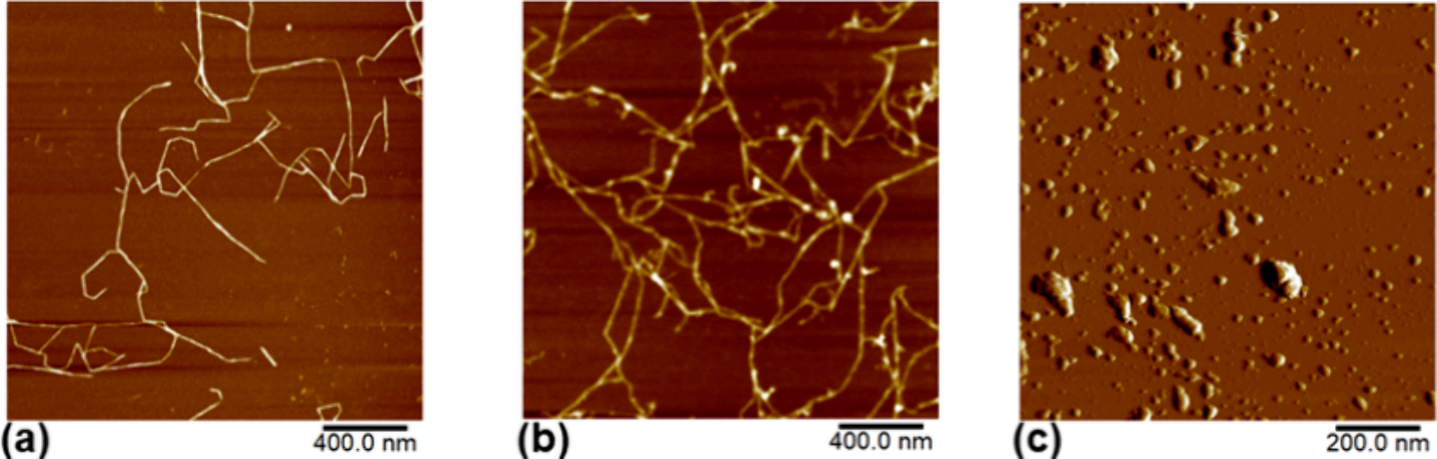
Atomic force microscopy height images of TOCNF (a) and PCNF (b) on a mica substrate using a diluted suspension and (c) peak force error of BSA protein in liquid media.

As shown in Figure 2a,b, where the micrographs of TOCNF and PCNF are displayed, the two types of functionalized nanocellulose structures are linear and homogeneous, with an average diameter of 2 ± 0.5 nm independently of the functional groups added. Their spatial organization as individual nanofibers is essentially due to their high surface charge, which keeps them apart. Concerning the BSA protein in Figure 1c, the AFM image shows a distribution on the mica substrate with an average size of 37.60 ± 8.36 nm, with some distinct aggregates. The XRD pattern and FTIR spectra are shown in Figure S1. Conductivity is shown in Figure S2. The zeta potential at a pH range of 2.27–10.95 in water and phosphate saline solutions is shown in Figure S3. Results from the characterization performed are summarized in Table 1.

**Table 1:** Characteristics of the Nanofibers TOCNF and PCNF, in addition to BSA

	TOCNF	PCNF	BSA	references
crystallinity index (%)	48.14	48.33		refs (46), (33, 47, 48), and (49)
charge content	1 mmol/g	3 mmol/g	+16 at pH = 3.92	
degree of substitution (DS)	0.16	0.33		ref (50)
FTIR	1601 cm^–1^, assigned to the C=O stretching of sodium-carboxyl groups	1216, 913, and 827 cm^–1^ corresponding to P=O, P–OH, and P–O–C, respectively	1642 and 1522 cm^–1^, ascribed to amide I C=O stretching and amide II C–N stretching and N–H bending modes, respectively	refs (51), (11, 12, 33, 52, 53), (54), and (55)

**Figure 3:**
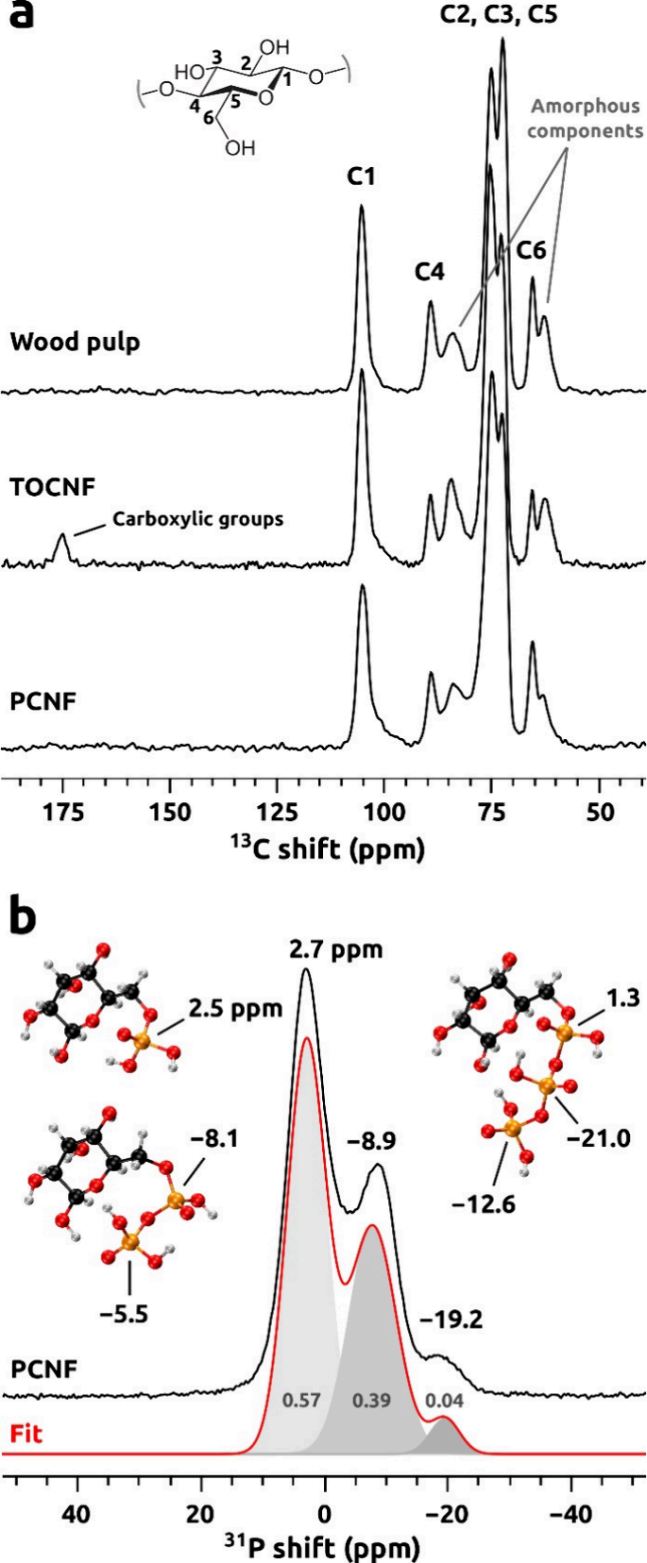
NMR analysis of wood pulp, TOCNF, and PCNF samples. (a) ^13^C CPMAS NMR spectra of wood pulp, TOCNF, and PCNF samples with corresponding links between the glucose carbon position and spectral characteristics. (b) ^31^P MAS NMR spectrum of the PCNF sample shown together with signal deconvolution (integrals given in gray) and models of hypothetical (poly)phosphate groups with the DFT-calculated ^31^P NMR shifts.

Solid-state NMR spectra were collected to gain further structural and chemical insights into the different nanocellulosic materials (Figure 3). The ^13^C CPMAS NMR spectra of the wood pulp (reference material) were compared to the chemically modified celluloses (TOCNF and PCNF). These showed similar features and revealed all characteristic signals of carbon atoms in cellulose in the chemical shift range of 60–110 ppm (Figure 3). In the TOCNF spectrum, an additional signal from carboxylic groups was observed at 175 ppm, which confirms the successful chemical modification. Indeed, significant differences between TOCNFs and the reference material in the signals of the C6 and C4 carbon atoms (and their counterparts in amorphous cellulose components) were detected. It confirms that the chemical modification occurred at the C6 carbon atoms.

In contrast, the PCNF sample spectrum was more similar to the reference material. Thus, to gain insights into chemical modifications in this sample, the ^31^P MAS NMR spectrum was collected (Figure 3b). Three relatively broad resonances were observed, with the peak maxima centered at 2.7, −8.9, and −19.2 ppm. These signals were assigned to phosphorus atoms involved in cellulose mono-, di-, and triphosphates, respectively, as suggested by the DFT calculations of ^31^P NMR chemical shifts carried out for the corresponding models (Figure 3b). The amount of triphosphates linked to nanocellulose was small, as shown by the small signal integral at −19.2 ppm (4% out of the total ^31^P signal intensity), whereas the diphosphate amount was very high. Comparing the integrals of the signals centered at 2.7 and −8.9 ppm revealed that the mono/diphosphate percentage ratio of phosphorus atoms involved in the material was approximately 60/40.

### AFM Probe Functionalization

Chemical modification of the AFM probes using (3-aminopropyl)triethoxysilane (APTES) was performed as previously reported,^28,34^ with some adjustments. These specifically included the silanization process that occurred in an inert atmosphere, whereby the ethoxy group of APTES reacted with the −OH group present on the surface of the tip, forming amine moieties. APTES-modified AFM probes were then utilized to anchor the nanocellulose fibers, resulting in a stable probe from the bonding between the nanocellulose and amine group, as was shown in a previous study.^28^ The functional groups of nanocellulose exposed to the side of the tips were not available for further interactions because they participated in binding with the amine group of the tip. However, the nanocellulose functional groups exposed on the other side remained available for further interaction. APTES-modified mica was also employed to covalently bond the BSA protein by reacting the amine groups with glutaraldehyde via the Schiff base formation between the amine group of either the APTES or the protein N-termini and aliphatic-NH_2_ groups and glutaraldehyde.^56^

**Figure 4:**
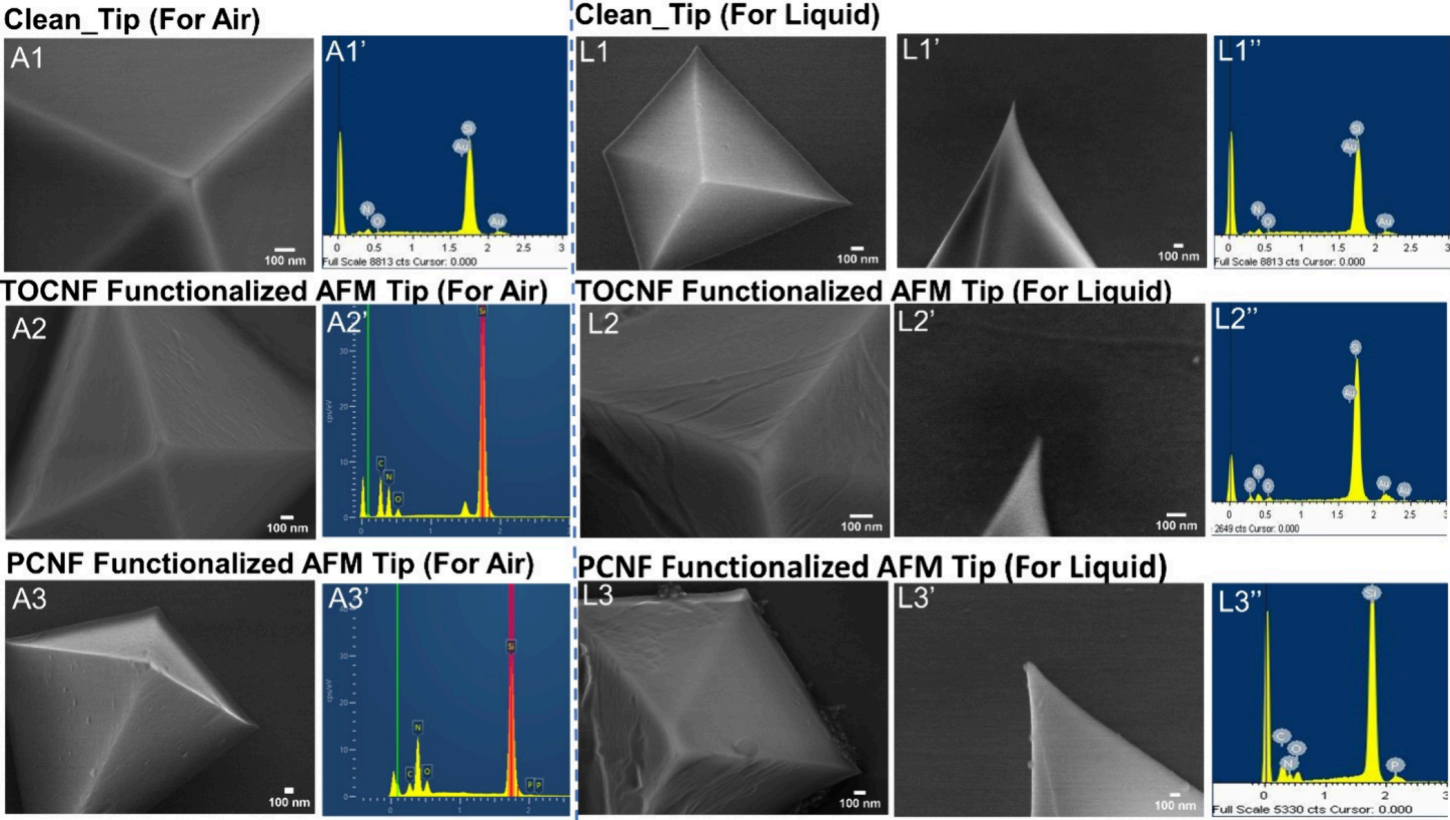
Scanning electron microscopy and energy-dispersive X-ray spectroscopy of AFM probes used in air and liquid measurements, (A1/A1’) clean-tip SEM and its EDS spectrum, (A2/A2’) SEM and EDS of the TOCNF-modified tip used in air force measurement, and (A3/A3′) SEM and EDS spectrum of the PCNF-modified tip used in air force measurement. The panel on the right shows the tips used in liquid force measurement (L1/L1’/L1”) corresponding to the clean tip, with the (L1’) side view for tip radius measurement, (L2/L2’/L2”) depicting the TOCNF-modified tip; (L3/L3′/L3”) the PCNF-modified tip.

The AFM probe’s condition and functionalization were verified using SEM after washing the nanocellulose-coated tip with water to remove the unbound excess. The electron micrographs indicated that the probes were covered with a rough nanometric layer of nanocellulose compared to the uncoated probes (Figure 4). Moreover, the probes had uniform sizes and shapes, which suggested that the contact area between the probes and the substrate was consistent during the force measurements. Additionally, EDS analysis provided information about the probes’ elemental composition and coating. The image of the clean tip is shown in Figure 4A1/L1, and its spectrum confirmed the anticipated presence of silicon, nitrogen, and oxygen resulting from the oxidation of silicon nitride surfaces under ambient conditions (Figure 4A1’/L1”). The probes modified with TOCNFs, as depicted in Figure 4A2/L2, presented an EDS spectrum with increased peak intensity for carbon, nitrogen, and silicon nitride (Figure 4A2’/L2”). In contrast, the probes coated with phosphorylated cellulose nanofibers (Figure 4A3/L3) showed in their spectrum the presence of the phosphorus element in the functionalized nanofibers (Figure 4A3′/L3”). The SEM images’ side views (Figure 4L1’/L2’/L3'). Were used to measure the probe tip radius, which was then employed to normalize the force measurements.

### Fluorescence Microscopy Imaging of the AFM Probes

**Figure 5:**
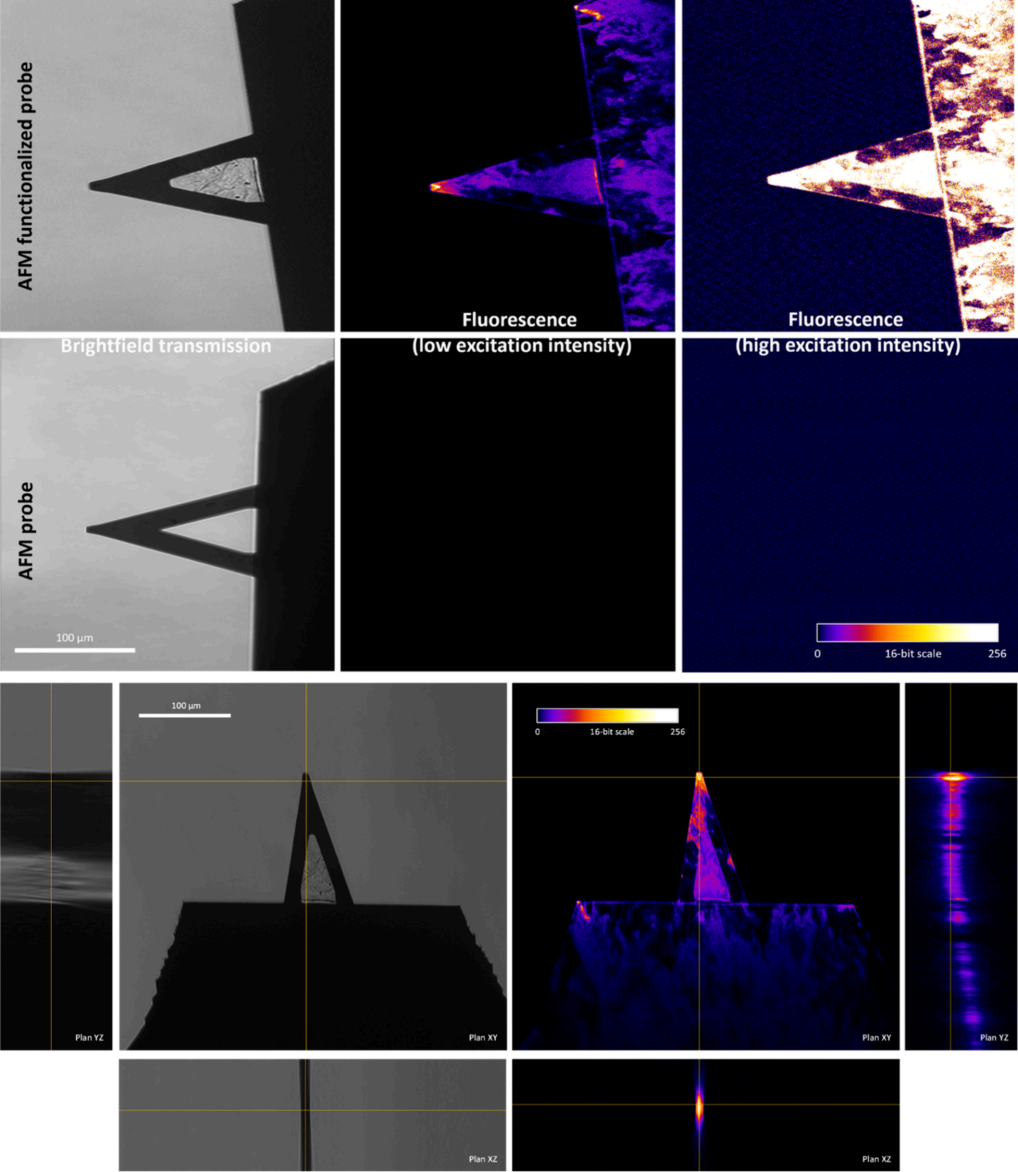
Comparison between the coating of AFM probes with rhodamine B-linked TOCNFs. Orthogonal image view across the AFM-functionalized probe in bright-field transmission and fluorescence. Note that the fluorescence intensity is represented with artificial colors [488 nm excitation and a long-pass emission of >504 nm]. Yellow crosshair lines indicate the spatial position in plane *XY* of the sectioning in *XZ* and *YZ*.

AFM probe coating was then confirmed using TOCNFs further functionalized with rhodamine B fluorophores and confocal fluorescence imaging. Comparison between probes coated with nanocellulose labeled or not with rhodamine B showed that, independently of the detection sensitivity levels, no fluorescence signals could be detected for probes coated with unlabeled nanocellulose (Figure 5). Fluorescence emission spectra, with maxima at 572–580 nm, under 488 nm excitation of nanocellulose in an isolated form or coated onto AFM probes, confirmed the specific presence of the rhodamine B fluorophore. Spatial distribution analyses using optical sectioning revealed that most of the coating signal was located at the AFM probe tip, further confirming the use of such coated AFM probes for force measurements.

### AFM Force Measurements in Air

**Figure 6:**
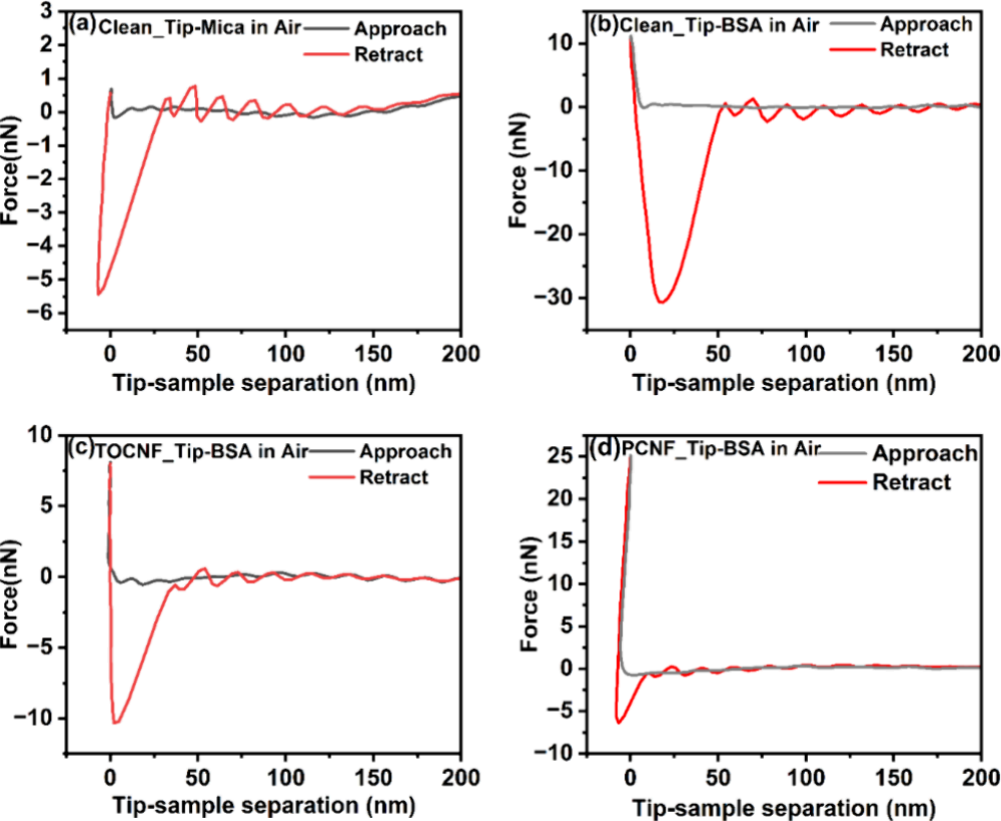
Representative force–distance curves in air of (a) clean tip and clean mica, (b) clean tip and BSA, (c) TOCNF-functionalized tip and BSA, and (d) PCNF-functionalized and tip-BSA. Note that the minima in the retract curve represent the adhesion force.

On a clean muscovite mica, with a surface roughness of about 0.101 nm, determined from the AFM image, the average adhesion force with an uncoated tip normalized to its radius was 0.14 ± 0.04 nN/nm (Figure 6a), which primarily resulted from a combination of electrostatic force, capillary force, and van der Waals force with the most important contribution from the capillary force due to the capillary condensation of water at ambient conditions.^57^ This adhesion force was affected by the surface energies of the substrate, tip, surface roughness, and humidity in the case of a hydrophilic substrate.^58−60^ The force–distance curve depicted in Figure 6b, during the approach cycle, indicated the tip bent due to attraction by the surface at a distance of around 7.5 nm.

During the retraction cycle, the adhesion force was 2.4 ± 0.4 nN/nm, which is 17.14 times higher than the substrate alone, given that the surface was rougher with an average roughness of 3.20 nm. This result indicated the affinity of the protein for the silicon nitride surface. In contrast, the uses of the AFM tip functionalized with TOCNFs and PCNFs showed a reduced magnitude of the adhesion force to BSA compared with the bare tip, which indicated that the protein interacted more strongly with the clean tip than the functionalized one. However, a weak adhesion force was still detected between BSA and TOCNFs, with a normalized adhesion force of 0.22 ± 0.09 nN/nm (Figure 6c). These results suggest a weak interaction between the hydroxyl and carboxyl groups of TOCNFs and the protein. Similar results were also observed with the PCNF-coated tips, whereby the mean normalized adhesion force decreased to 0.14 ± 0.06 nN/nm (Figure 6d). We can speculate that this even weaker force was possibly due to the higher number of charged phosphate groups interacting unfavorably with the negatively charged groups of the protein. The force–distance curve did not indicate clear evidence for protein unfolding, as it is free from the jumps during the retraction curve.^61^

### AFM Force Measurements in Liquid

**Figure 7:**
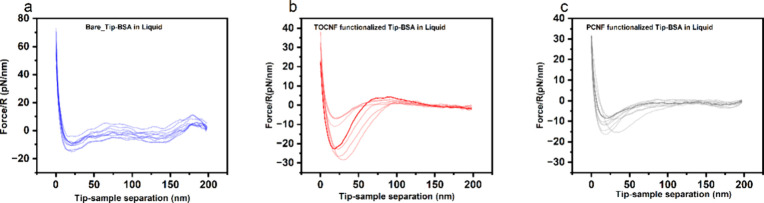
Collection of normalized force–distance curves by the tip radius of (a) clean tip and BSA, (c) TOCNF-functionalized tip and BSA, and (c) PCNF-functionalized tip and BSA in PS as a liquid medium. Note that the curves are in the retraction mode, and adhesion force is at the minimum.

The interaction between BSA and nanocellulose was evaluated in a phosphate saline (PS) solution to mimic its behavior in a quasi-native environment. These measurements at a fixed pH of 3.92 could show the charge differences from those at pH 7, which show no interactions between BSA and nanocellulose.^20^ The collection of normalized force–distance curves in the retraction mode is shown in Figure 7, which are derived from the images (see Figure 2c) by generating a force–distance curve where the protein exists on the substrate. The tip velocity was fixed at 0.2 μm/s during the measurement. During the interaction between the bare tip and BSA-coated mica, a normalized adhesion force of approximately 10.83 ± 2.28 pN/nm was recorded (as shown in Figure 6a). This was followed by multiple jumps in the distance of roughly 0.016 nm each, possibly due to the stretching of the protein.^62,63^ In addition, the TOCNF-functionalized tip presented a value of the normalized adhesion force of 17.87 ± 8.58 pN/nm (Figure 7b). The AFM probe functionalized with PCNFs showed a normalized adhesion force of 11.70 ± 2.97 pN/nm, which is in the same range as TOCNFs (Figure 7c). Previous work reporting the thermodynamics of interactions on positively charged cellulose nanocrystals showed that the adsorption capacity is linearly dependent on the surface density. Still, on the other hand, the binding strength (a measure of affinity) decreased with increasing surface density.^64^ The authors explained this by taking into account the steric hindrance for the adsorbate, which is higher for nanocrystals with a higher grafting density, and this could also apply to this system, as the charge density of PCNFs is three times higher than for TOCNFs (see Table 1). Based on the results of our experiments, we can conclude that both TOCNF and PCNF nanomaterials exhibited low adhesion forces, making them promising candidates as a nonadsorbing substrate for BSA.

### Reactive Molecular Dynamics Simulation

**Figure 8:**
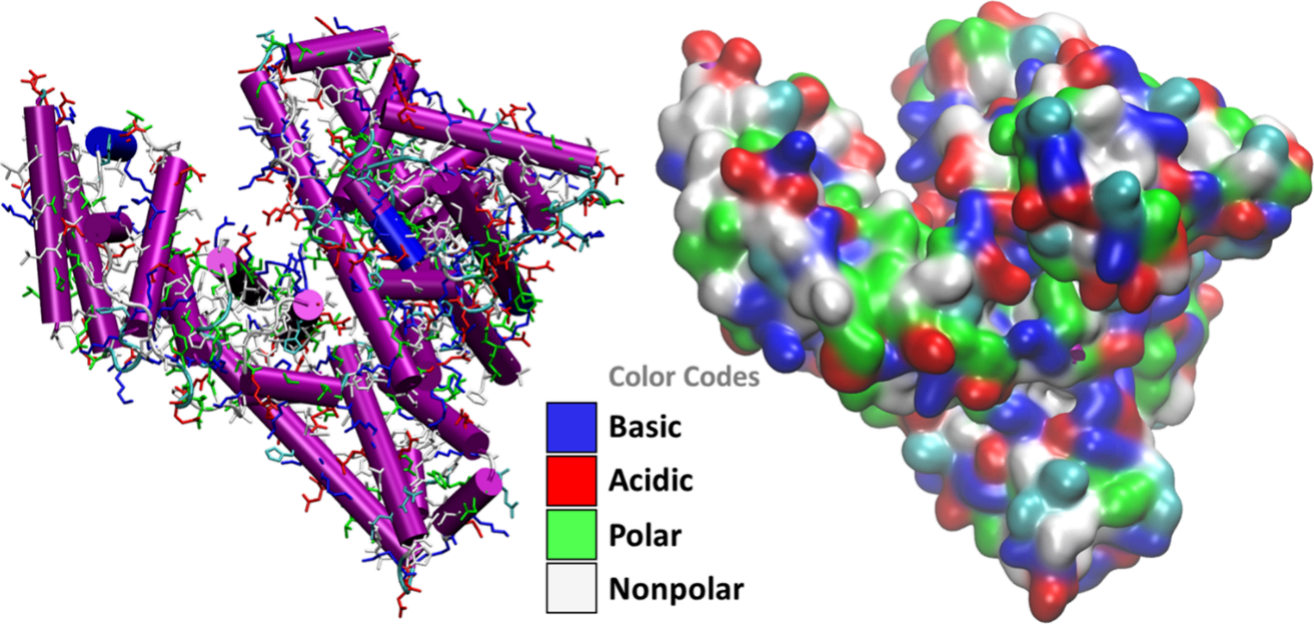
BSA structure from the Protein Data Bank (PDB) displaying the helical motifs and the character of the amino acids on the solvent-accessible surface. The basic residues are mainly protonated lysine.

The results of the reactive molecular dynamics simulations were used to support the experimental description. From the analysis of the sampled data, it was found that the assembled fibers, randomly functionalized with phosphate and carboxyl groups covered with water and counterions, maintained their hydrophilic character and, thus, the protein’s propensity to adsorb on the nanocellulose surface through basic and acidic residues. These were distributed almost uniformly on the BSA surface (Figure 8) and, during the motion of the protein toward the interface, could elongate their side chains and form salt bridges and hydrogen bonds not only with the adsorbed water layers but also with the cellulose head groups.

**Figure 9:**
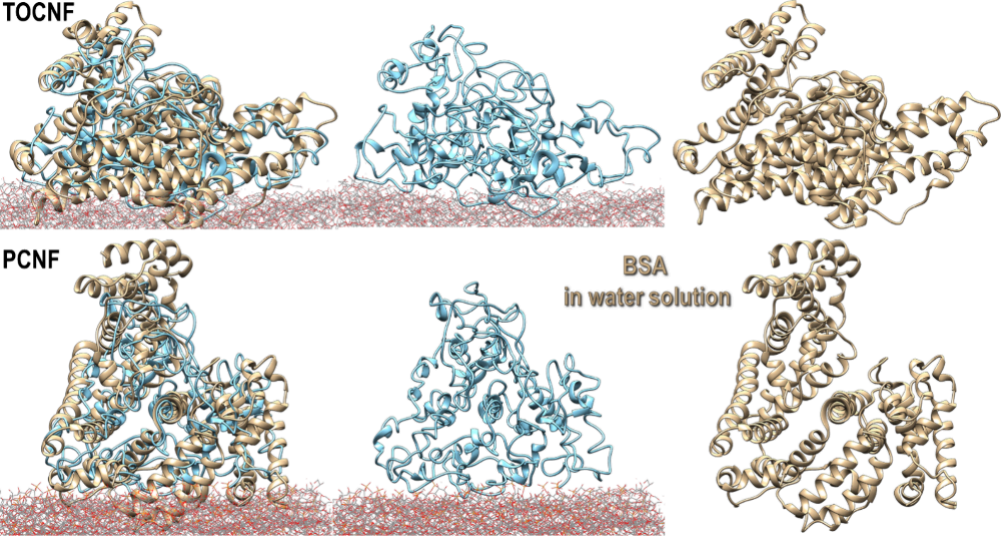
Adsorbed structures of BSA (best interaction energy per atom) on TOCNF (top) and PCNF (bottom) superimposed on the trace (Cα: best RMSD fit) of an average equilibrium structure of the protein in water solution obtained through classical nonreactive.

Indeed, the approach of BSA to the cellulose interface determined a relocation of Na^+^ ions and water molecules and the formation of large exposed areas where direct interactions between BSA and cellulose reinforced their mitigated binding (appearance of active anchoring points of complementary binders). This effect is evident in Figure S8, where the TOCNF and PCNF surfaces with the remaining waters and ions after BSA adsorption are displayed (Figure S8c,f) together with the regions occupied by the BSA contact residues (Figure S8a,d). Visual examination of all adsorbed conformations of BSA (Figure S5) and the root-mean-square deviation of the trace (C_α_) values from the original solvated structure (Table S3) revealed that the deposited geometries were partially unfolded. This partial denaturation at the cellulose interface was due to the breaking of the hydrogen bonds in the protein’s secondary structure that happened upon binding to the interface (the localization of protein helices is indicated in Tables S5–S7). The adsorbed BSA conformations examined here were more contracted than structures in solution because we removed the surrounding solvent to speed up the calculations. Still, we found that more than 20% of their solvent-accessible surface (SAS) was on top of the cellulose matrices (Table S3). In these contact regions, most of the LYS residues interacting with the surface released their hydrogens to the functionalizing layers (tables in Figure S6), and the most stable adsorptions (Figure 9 and Figure S8) seemed dominated by the negatively charged amino acids. It turned out that BSA more favorably interacted with TOCNF by about 1.5 kcal/mol per atom than with PCNF but also deviated less from the original structure by about 1.2 Å and had the highest number of amino acids in contact with the cellulose surface (26% more than in the other case). To calculate the adsorption strength of the two cellulose supports, we determined the number of adsorbed protein residues on the surface of each model (Table S3) and plotted the resulting interaction energies against this number (Figure S7). Comparing the slopes of each linear fitting, it is apparent that the TOCNF has a slightly higher interaction energy, even though we recognized that the differences are subtle because of the celluloses and BSA hydrophilicity. In fact, it was impossible to identify definite preferences and discriminate among different protein orientations (Figure S5). A closer view of the interface for the stronger interaction energy models of TOCNF and PCNF is shown in Figure S8. However, many arrangements could be highly probable and present simultaneously on the cellulose interfaces. The simulations showed that the main protein deformations originated from the constraining action of the cellulose-binding sites, which reduced the flexibility of the BSA chains and determined the loss of most of the intramolecular hydrogen bonds. An earlier investigation^64^ reported a similar mechanism for the binding of BSA to positively charged CNCs, suggesting an entropy-driven mechanism, which was associated with the release of surface-structured water and counterions from the nanocellulose surface. It was also demonstrated that the BSA had to unfold to become attached to specific binding sites. A similar mechanism was reported for the adsorption at nanocellulose surfaces of ions of opposite charge and found that the entropy increase that arose from the release of surface-structured water molecules and counterions was the major contribution to the free energy of adsorption.^14^

In connection with the simulations, we could speculate that the difference between the two nanocelluloses could be due to the variety of balanced/unbalanced interactions between the protein residues and the head groups of the functionalizing chains, sometimes mediated by the interposed water molecules and counterions. The examination of the dynamics of the systems revealed frequent exchanges of hydrogen atoms in different amounts of adsorbed water molecules/counterions. We noticed that in the case of PCNF, we could have a conspicuous concentration of metal ions, partially solvated, preferentially bound to the phosphates, which displaced the direct protein–cellulose connections. The preference for TOCNF could be due to carboxyl moieties, which are more prone to forming salt bridges with the amine head groups (with variable protonation states depending on the surrounding species) of the proteins close to the interface. The local pH and, in general, the pH of the environment have a pronounced effect on the competition between the protein connections to the surface and the metal ions connected to the charged phosphates oriented toward the solution.

## Conclusions

In this work, we have determined the interactions between functionalized cellulose nanofibers and the BSA protein using a concerted multiscale strategy based on atomic force microscopy and reactive molecular dynamics simulations. We successfully functionalized the AFM probes with cellulose nanofibers and designed a new method based on fluorescence to visualize and validate the nanocellulose attachment to the tip. With the AFM data, we disclosed lower adhesion forces associated with two differently functionalized nanocelluloses and the protein.

We have concluded that when evaluating the interactions between functionalized cellulose and BSA, two different pictures should be taken into account, namely, long- and short-range interactions. The first one is the driving force of the adsorption (complementarity of the molecular electrostatic potential), which can be explained through the putative charge state of the systems determined by the pH of the environment (systems far apart), and is obtained from the experimental work. The second one, based on the simulations, shows the direct connection of the two systems when they are very close to each other, which is regulated by local intermolecular interactions and a reorganization/definition of the charge state of the side chains of both systems (i.e., protein and fibers). The atomic-level description of the interface between the assembled randomly functionalized cellulose fibers (covered with water and counterions) and the protein, provided by the RMD simulations, suggested that the BSA adsorption process (chain contacts) could take place through many different regions of the protein surface due to the presence of both basic and acidic residues, which were almost uniformly distributed on the BSA surface (sparse patches visible in Figure 6). The protein/cellulose binding is very subtle and cannot be characterized effectively either experimentally or computationally due to the vast statistics needed.

The results found with all our techniques prove that the type, concentration, and location of nanocellulose surface functionality play a crucial role in the interactions of these materials with proteins, solvents, and ions. Using the simulations, we showed that cellulose nanofibers and BSA interaction could induce protein unfolding. Given all these findings, we could conclude that the generalization of the binding mechanisms as the interaction between big monocharged objects is an oversimplification of complex actions where subtle mechanisms at the atomic level take place to regulate the whole dynamics of adsorption.

In the broader context of nanotechnology and biophysics, this research contributes to the fundamental knowledge of nanomaterial behavior and its implications for diverse applications. It holds immense promise for shaping the future of materials science and biotechnology, underscoring the importance of interdisciplinary research.

## Supporting Information Available

Characterization of cellulose nanofibers in terms of FTIR, TGA, XRD, and conductometric titration; zeta potential of all suspensions and BSA; supporting data related to the modeling part (PDF)
