# Supplementary material for: Nanocellulose-Bovine Serum Albumin Interactions in an Aqueous Medium: Investigations Using In Situ Nanocolloidal Probe Microscopy and Reactive Molecular Dynamics Simulations
Source: Biomacromolecules. 2024 May 28;25(6):3703–14. doi: 10.1021/acs.biomac.4c00264 (PMC11170956; doi:10.1021/acs.biomac.4c00264)
Supplement: Supplementary file 1 — bm4c00264_si_001.pdf [file bm4c00264_si_001.pdf]

Supporting information

Nanocellulose-Bovine Serum Albumin Interactions in Aqueous Medium: Investigations Using *in-situ* Nanocolloidal Probe Microscopy and Reactive Molecular Dynamics Simulations

Houssine Khalili[a], Susanna Monti[b], Edouard Pesquet[c], Aleksander Jaworski[a], Salvatore Lombardo[a], and Aji P Mathew[a,*].

[a]Department of Materials and Environmental Chemistry, Stockholm University, 10691 Stockholm, Sweden.

[b]CNR-ICCOM – Institute of Chemistry of Organometallic Compounds, via Moruzzi 1, 56124 Pisa, Italy.

[c] Department of Ecology, Environment and Plant Sciences, Stockholm University, 10691 Stockholm, Sweden.

Email Address of the Authors:

Houssine Khalili: houssine.khalili@mmk.su.se

Susanna Monti: sapeptides@gmail.com ; susanna.monti@cnr.it

Edouard Pesquest: edouard.pesquet@su.se

Aleksander Jaworski: aleksander.jaworski@mmk.su.se

Salvatore Lombardo: salvatore.lombardo@mmk.su.se

* Corresponding author (Aji P Mathew): aji.mathew@mmk.su.se

S1. FTIR, TGA/DTG, and XRD characterization of nanocellulsoe fibers.

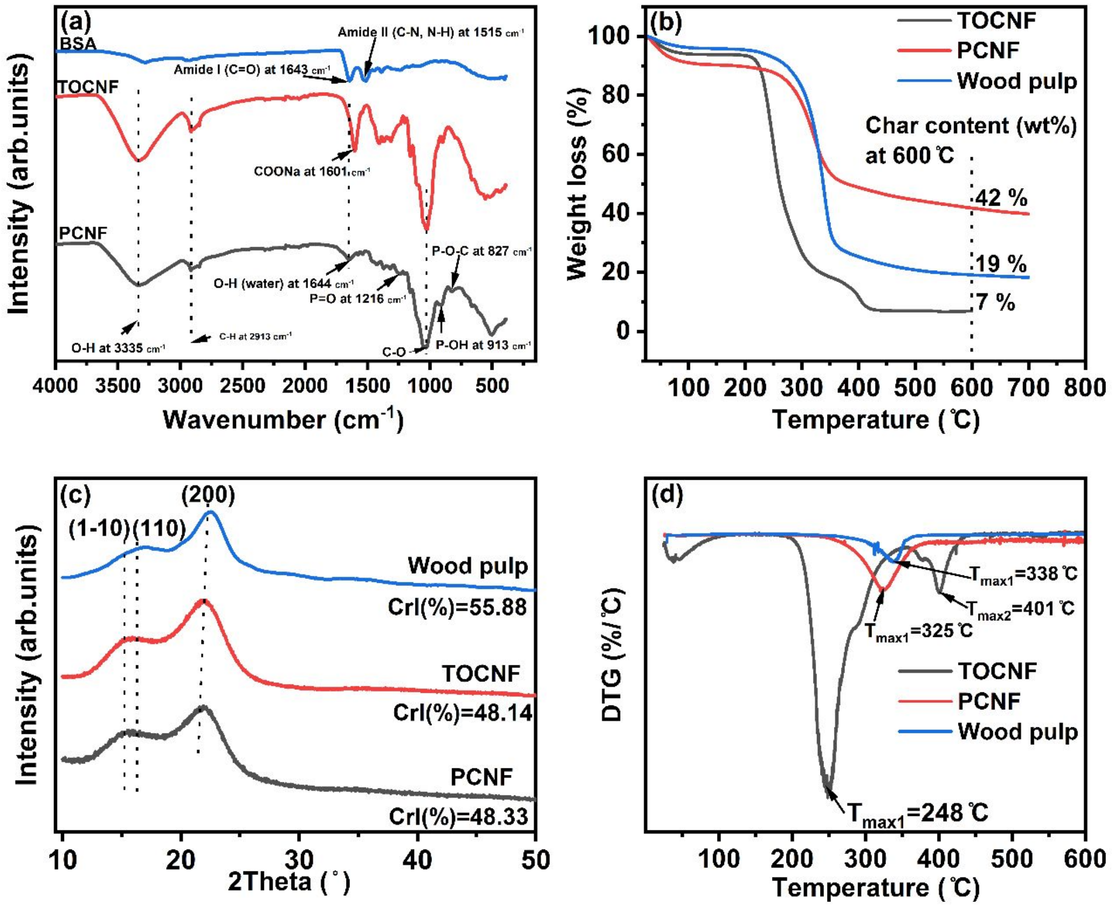

Figure S1. (a) BSA, TOCNF, and PCNF FTIR spectra, (b)/(d) TGA and DTG curves for all cellulosic materials, and (c) XRD patterns of TOCNF, PCNF, and wood pulp.

S2. Charge content (CC) of PCNF and TOCNF.

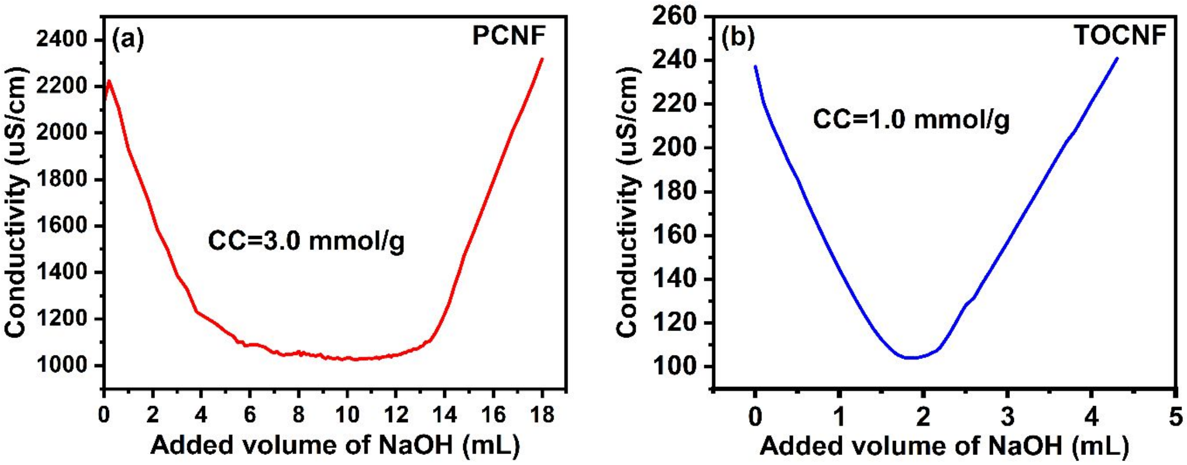

Figure S2. Conductometric titration results of phosphorylated cellulose nanofibers (a) and TEMPO-oxidized cellulose nanofibers (b).

S3. Zeta potential of BSA, TOCNF, and PCNF in water and PS at different pH.

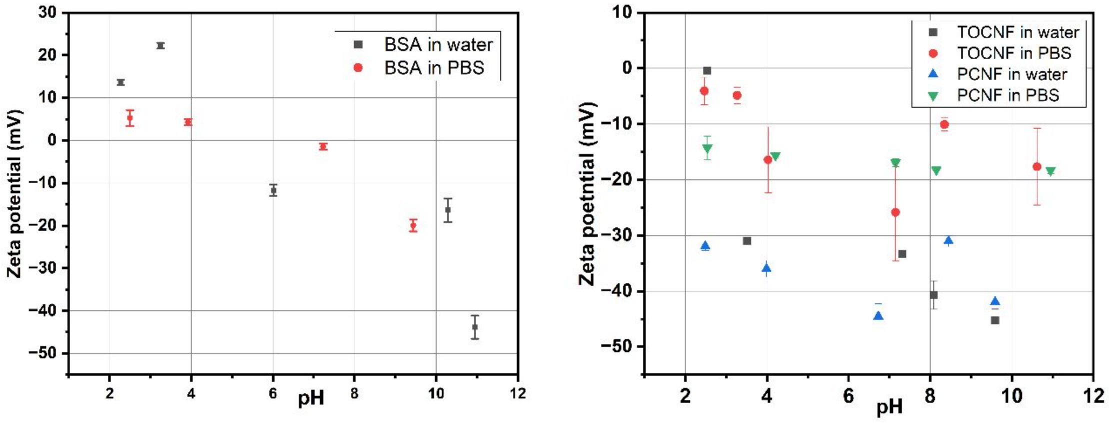

Figure S3. Zeta potential results of BSA, TOCNF, and PCNF.

MODELING – SUPPORTING DATA

Table S1. Number of basic (blue), acidic (red), and neutral (black) amino acids of BSA at pH 7.0 and 3.92.

Res.	pH 7.0	pH 3.92
ARG	23	23
ARN	0	0
LYS	59	59
LYN	0	0
HIP	7	15
HIE	8	2
HID	2	0
ASP	39	36
ASH	0	3
GLU	59	45
GLH	0	14
CYS	1	1
CYM	0	0
CYX	34	34
charge	-9	+16

HIE and HID are neutral histidines with epsilon or delta protonation, CYX are cysteines in S-S bridges

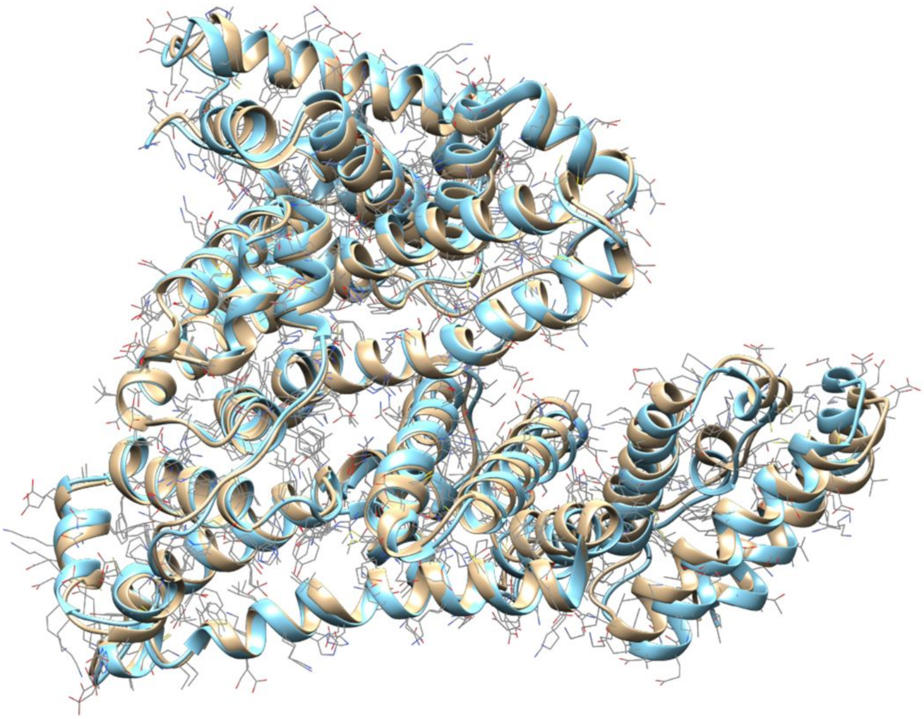

Figure S4.Best superimposed structures of the BSA original model (PDB: 3v03 – light brown) and the average structure of the molecule obtained from the MD simulations in water solution (cyan). The root mean square deviation of the trace atoms (C_{α}) is approximately 1.2 Å. Hydrogens have been undisplayed.

Table S2. Amino acids sequence of BSA. HIP is the protonated histidine, whereas HIE and HID are neutral histidines with epsilon or delta protonation; CYX are cysteines in S-S bridges. ASH and GLH are the protonated aspartic and glutamic acids, respectively.

Res #	pH 7.0	pH 3.92
1	HIP	HIP
2	LYS	LYS
3	SER	SER
4	GLU	GLU
5	ILE	ILE
6	ALA	ALA
7	HIP	HIP
8	ARG	ARG
9	PHE	PHE
10	LYS	LYS
11	**ASP**	**ASH**
12	LEU	LEU
13	GLY	GLY
14	GLU	GLU
15	GLU	GLU
16	HIP	HIP
17	PHE	PHE
18	LYS	LYS
19	GLY	GLY
20	LEU	LEU
21	VAL	VAL
22	LEU	LEU
23	ILE	ILE
24	ALA	ALA
25	PHE	PHE
26	SER	SER
27	GLN	GLN
28	TYR	TYR
29	LEU	LEU
30	GLN	GLN
31	GLN	GLN
32	CYS	CYS
33	PRO	PRO
34	PHE	PHE
35	ASP	ASP
36	GLU	GLU
37	**HID**	**HIP**
38	VAL	VAL
39	LYS	LYS
40	LEU	LEU
41	VAL	VAL
42	ASN	ASN
43	**GLU**	**GLH**
44	LEU	LEU
45	THR	THR
46	GLU	GLU
47	PHE	PHE
48	ALA	ALA
49	LYS	LYS
50	THR	THR
51	CYX	CYX
52	VAL	VAL
53	ALA	ALA
54	ASP	ASP
55	GLU	GLU
56	SER	SER
57	**HIE**	**HIP**
58	ALA	ALA
59	GLY	GLY
60	CYX	CYX
61	GLU	GLU
62	LYS	LYS
63	SER	SER
64	LEU	LEU
65	HIP	HIP
66	THR	THR
67	LEU	LEU
68	PHE	PHE
69	GLY	GLY
70	ASP	ASP
71	GLU	GLU
72	LEU	LEU
73	CYX	CYX
74	LYS	LYS
75	VAL	VAL
76	ALA	ALA
77	SER	SER
78	LEU	LEU
79	ARG	ARG
80	**GLU**	**GLH**
81	THR	THR
82	TYR	TYR
83	GLY	GLY
84	ASP	ASP
85	MET	MET
86	ALA	ALA
87	ASP	ASP
88	CYX	CYX
89	CYX	CYX
90	**GLU**	**GLH**
91	LYS	LYS
92	GLN	GLN
93	GLU	GLU
94	PRO	PRO
95	GLU	GLU
96	ARG	ARG
97	ASN	ASN
98	GLU	GLU
99	CYX	CYX
100	PHE	PHE
101	LEU	LEU
102	SER	SER
103	HIP	HIP
104	LYS	LYS
105	ASP	ASP
106	ASP	ASP
107	SER	SER
108	PRO	PRO
109	ASP	ASP
110	LEU	LEU
111	PRO	PRO
112	LYS	LYS
113	LEU	LEU
114	LYS	LYS
115	PRO	PRO
116	ASP	ASP
117	PRO	PRO
118	ASN	ASN
119	THR	THR
120	LEU	LEU
121	CYX	CYX
122	ASP	ASP
123	**GLU**	**GLH**
124	PHE	PHE
125	LYS	LYS
126	ALA	ALA
127	ASP	ASP
128	**GLU**	**GLH**
129	LYS	LYS
130	LYS	LYS
131	PHE	PHE
132	TRP	TRP
133	GLY	GLY
134	LYS	LYS
135	TYR	TYR
136	LEU	LEU
137	TYR	TYR
138	GLU	GLU
139	ILE	ILE
140	ALA	ALA
141	ARG	ARG
142	ARG	ARG
143	HIE	HIE
144	PRO	PRO
145	TYR	TYR
146	PHE	PHE
147	TYR	TYR
148	ALA	ALA
149	PRO	PRO
150	GLU	GLU
151	LEU	LEU
152	LEU	LEU
153	TYR	TYR
154	TYR	TYR
155	ALA	ALA
156	ASN	ASN
157	LYS	LYS
158	TYR	TYR
159	ASN	ASN
160	GLY	GLY
161	VAL	VAL
162	PHE	PHE
163	GLN	GLN
164	GLU	GLU
165	CYX	CYX
166	CYX	CYX
167	GLN	GLN
168	ALA	ALA
169	**GLU**	**GLH**
170	ASP	ASP
171	LYS	LYS
172	GLY	GLY
173	ALA	ALA
174	CYX	CYX
175	LEU	LEU
176	LEU	LEU
177	PRO	PRO
178	LYS	LYS
179	ILE	ILE
180	GLU	GLU
181	THR	THR
182	MET	MET
183	ARG	ARG
184	GLU	GLU
185	LYS	LYS
186	VAL	VAL
187	LEU	LEU
188	THR	THR
189	SER	SER
190	SER	SER
191	ALA	ALA
192	ARG	ARG
193	GLN	GLN
194	ARG	ARG
195	LEU	LEU
196	ARG	ARG
197	CYX	CYX
198	ALA	ALA
199	SER	SER
200	ILE	ILE
201	GLN	GLN
202	LYS	LYS
203	PHE	PHE
204	GLY	GLY
205	GLU	GLU
206	ARG	ARG

**207**	ALA	ALA
**208**	LEU	LEU
**209**	LYS	LYS
**210**	ALA	ALA
**211**	TRP	TRP
**212**	SER	SER
**213**	VAL	VAL
**214**	ALA	ALA
**215**	ARG	ARG
**216**	LEU	LEU
**217**	SER	SER
**218**	GLN	GLN
**219**	LYS	LYS
**220**	PHE	PHE
**221**	PRO	PRO
**222**	LYS	LYS
**223**	ALA	ALA
**224**	**GLU**	**GLH**
**225**	PHE	PHE
**226**	VAL	VAL
**227**	GLU	GLU
**228**	VAL	VAL
**229**	THR	THR
**230**	LYS	LYS
**231**	LEU	LEU
**232**	VAL	VAL
**233**	THR	THR
**234**	ASP	ASP
**235**	LEU	LEU
**236**	THR	THR
**237**	LYS	LYS
**238**	VAL	VAL
**239**	HIE	HIE
**240**	LYS	LYS
**241**	GLU	GLU
**242**	CYX	CYX
**243**	CYX	CYX
**244**	HIE	HIP
**245**	GLY	GLY
**246**	ASP	ASP
**247**	LEU	LEU
**248**	LEU	LEU
**249**	**GLU**	**GLH**
**250**	CYX	CYX
**251**	ALA	ALA
**252**	ASP	ASP
**253**	ASP	ASP
**254**	ARG	ARG
**255**	ALA	ALA
**256**	**ASP**	**ASH**
**257**	LEU	LEU
**258**	ALA	ALA
**259**	LYS	LYS
**260**	TYR	TYR

**261**	ILE	ILE
**262**	CYX	CYX
**263**	ASP	ASP
**264**	ASN	ASN
**265**	GLN	GLN
**266**	ASP	ASP
**267**	THR	THR
**268**	ILE	ILE
**269**	SER	SER
**270**	SER	SER
**271**	LYS	LYS
**272**	LEU	LEU
**273**	LYS	LYS
**274**	**GLU**	**GLH**
**275**	CYX	CYX
**276**	CYX	CYX
**277**	ASP	ASP
**278**	LYS	LYS
**279**	PRO	PRO
**280**	LEU	LEU
**281**	LEU	LEU
**282**	GLU	GLU
**283**	LYS	LYS
**284**	SER	SER
**285**	**HIE**	**HIP**
**286**	CYX	CYX
**287**	ILE	ILE
**288**	ALA	ALA
**289**	GLU	GLU
**290**	VAL	VAL
**291**	GLU	GLU
**292**	LYS	LYS
**293**	ASP	ASP
**294**	ALA	ALA
**295**	ILE	ILE
**296**	PRO	PRO
**297**	**GLU**	**GLH**
**298**	ASN	ASN
**299**	LEU	LEU
**300**	PRO	PRO
**301**	PRO	PRO
**302**	LEU	LEU
**303**	THR	THR
**304**	ALA	ALA
**305**	**ASP**	**ASH**
**306**	PHE	PHE
**307**	ALA	ALA
**308**	GLU	GLU
**309**	ASP	ASP
**310**	LYS	LYS
**311**	ASP	ASP
**312**	VAL	VAL
**313**	CYX	CYX
**314**	LYS	LYS

**315**	ASN	ASN
**316**	TYR	TYR
**317**	GLN	GLN
**318**	**GLU**	**GLH**
**319**	ALA	ALA
**320**	LYS	LYS
**321**	ASP	ASP
**322**	ALA	ALA
**323**	PHE	PHE
**324**	LEU	LEU
**325**	GLY	GLY
**326**	SER	SER
**327**	PHE	PHE
**328**	LEU	LEU
**329**	TYR	TYR
**330**	GLU	GLU
**331**	TYR	TYR
**332**	SER	SER
**333**	ARG	ARG
**334**	ARG	ARG
**335**	HIP	HIP
**336**	PRO	PRO
**337**	GLU	GLU
**338**	TYR	TYR
**339**	ALA	ALA
**340**	VAL	VAL
**341**	SER	SER
**342**	VAL	VAL
**343**	LEU	LEU
**344**	LEU	LEU
**345**	ARG	ARG
**346**	LEU	LEU
**347**	ALA	ALA
**348**	LYS	LYS
**349**	GLU	GLU
**350**	TYR	TYR
**351**	GLU	GLU
**352**	ALA	ALA
**353**	THR	THR
**354**	LEU	LEU
**355**	**GLU**	**GLH**
**356**	**GLU**	**GLH**
**357**	CYX	CYX
**358**	CYX	CYX
**359**	ALA	ALA
**360**	LYS	LYS
**361**	ASP	ASP
**362**	ASP	ASP
**363**	PRO	PRO
**364**	HIP	HIP
**365**	ALA	ALA
**366**	CYX	CYX
**367**	TYR	TYR
**368**	SER	SER

**369**	THR	THR
**370**	VAL	VAL
**371**	PHE	PHE
**372**	ASP	ASP
**373**	LYS	LYS
**374**	LEU	LEU
**375**	LYS	LYS
**376**	**HID**	**HIP**
**377**	LEU	LEU
**378**	VAL	VAL
**379**	ASP	ASP
**380**	GLU	GLU
**381**	PRO	PRO
**382**	GLN	GLN
**383**	ASN	ASN
**384**	LEU	LEU
**385**	ILE	ILE
**386**	LYS	LYS
**387**	GLN	GLN
**388**	ASN	ASN
**389**	CYX	CYX
**390**	ASP	ASP
**391**	GLN	GLN
**392**	PHE	PHE
**393**	**GLU**	**GLH**
**394**	LYS	LYS
**395**	LEU	LEU
**396**	GLY	GLY
**397**	GLU	GLU
**398**	TYR	TYR
**399**	GLY	GLY
**400**	PHE	PHE
**401**	GLN	GLN
**402**	ASN	ASN
**403**	ALA	ALA
**404**	LEU	LEU
**405**	ILE	ILE
**406**	VAL	VAL
**407**	ARG	ARG
**408**	TYR	TYR
**409**	THR	THR
**410**	ARG	ARG
**411**	LYS	LYS
**412**	VAL	VAL
**413**	PRO	PRO
**414**	GLN	GLN
**415**	VAL	VAL
**416**	SER	SER
**417**	THR	THR
**418**	PRO	PRO
**419**	THR	THR
**420**	LEU	LEU
**421**	VAL	VAL
**422**	GLU	GLU

**423**	VAL	VAL
**424**	SER	SER
**425**	ARG	ARG
**426**	SER	SER
**427**	LEU	LEU
**428**	GLY	GLY
**429**	LYS	LYS
**430**	VAL	VAL
**431**	GLY	GLY
**432**	THR	THR
**433**	ARG	ARG
**434**	CYX	CYX
**435**	CYX	CYX
**436**	THR	THR
**437**	LYS	LYS
**438**	PRO	PRO
**439**	GLU	GLU
**440**	SER	SER
**441**	GLU	GLU
**442**	ARG	ARG
**443**	MET	MET
**444**	PRO	PRO
**445**	CYX	CYX
**446**	THR	THR
**447**	GLU	GLU
**448**	ASP	ASP
**449**	TYR	TYR
**450**	LEU	LEU
**451**	SER	SER
**452**	LEU	LEU
**453**	ILE	ILE
**454**	LEU	LEU
**455**	ASN	ASN
**456**	ARG	ARG
**457**	LEU	LEU
**458**	CYX	CYX
**459**	VAL	VAL
**460**	LEU	LEU
**461**	**HIE**	**HIP**
**462**	GLU	GLU

**463**	LYS	LYS
**464**	THR	THR
**465**	PRO	PRO
**466**	VAL	VAL
**467**	SER	SER
**468**	GLU	GLU
**469**	LYS	LYS
**470**	VAL	VAL
**471**	THR	THR
**472**	LYS	LYS
**473**	CYX	CYX
**474**	CYX	CYX
**475**	THR	THR
**476**	GLU	GLU
**477**	SER	SER
**478**	LEU	LEU
**479**	VAL	VAL
**480**	ASN	ASN
**481**	ARG	ARG
**482**	ARG	ARG
**483**	PRO	PRO
**484**	CYX	CYX
**485**	PHE	PHE
**486**	SER	SER
**487**	ALA	ALA
**488**	LEU	LEU
**489**	THR	THR
**490**	PRO	PRO
**491**	ASP	ASP
**492**	GLU	GLU
**493**	THR	THR
**494**	TYR	TYR
**495**	VAL	VAL
**496**	PRO	PRO
**497**	LYS	LYS
**498**	ALA	ALA
**499**	PHE	PHE
**500**	ASP	ASP
**501**	GLU	GLU
**502**	LYS	LYS

**503**	LEU	LEU
**504**	PHE	PHE
**505**	THR	THR
**506**	PHE	PHE
**507**	**HIE**	**HIP**
**508**	ALA	ALA
**509**	ASP	ASP
**510**	ILE	ILE
**511**	CYX	CYX
**512**	THR	THR
**513**	LEU	LEU
**514**	PRO	PRO
**515**	ASP	ASP
**516**	THR	THR
**517**	GLU	GLU
**518**	LYS	LYS
**519**	GLN	GLN
**520**	ILE	ILE
**521**	LYS	LYS
**522**	LYS	LYS
**523**	GLN	GLN
**524**	THR	THR
**525**	ALA	ALA
**526**	LEU	LEU
**527**	VAL	VAL
**528**	GLU	GLU
**529**	LEU	LEU
**530**	LEU	LEU
**531**	LYS	LYS
**532**	**HIE**	**HIP**
**533**	LYS	LYS
**534**	PRO	PRO
**535**	LYS	LYS
**536**	ALA	ALA
**537**	THR	THR
**538**	GLU	GLU
**539**	GLU	GLU
**540**	GLN	GLN
**541**	LEU	LEU
**542**	LYS	LYS

**543**	THR	THR
**544**	VAL	VAL
**545**	MET	MET
**546**	GLU	GLU
**547**	ASN	ASN
**548**	PHE	PHE
**549**	VAL	VAL
**550**	ALA	ALA
**551**	PHE	PHE
**552**	VAL	VAL
**553**	ASP	ASP
**554**	LYS	LYS
**555**	CYX	CYX
**556**	CYX	CYX
**557**	ALA	ALA
**558**	ALA	ALA
**559**	ASP	ASP
**560**	ASP	ASP
**561**	LYS	LYS
**562**	GLU	GLU
**563**	ALA	ALA
**564**	CYX	CYX
**565**	PHE	PHE
**566**	ALA	ALA
**567**	VAL	VAL
**568**	GLU	GLU
**569**	GLY	GLY
**570**	PRO	PRO
**571**	LYS	LYS
**572**	LEU	LEU
**573**	VAL	VAL
**574**	VAL	VAL
**575**	SER	SER
**576**	THR	THR
**577**	GLN	GLN
**578**	THR	THR
**579**	ALA	ALA
**580**	LEU	LEU
**581**	ALA	ALA

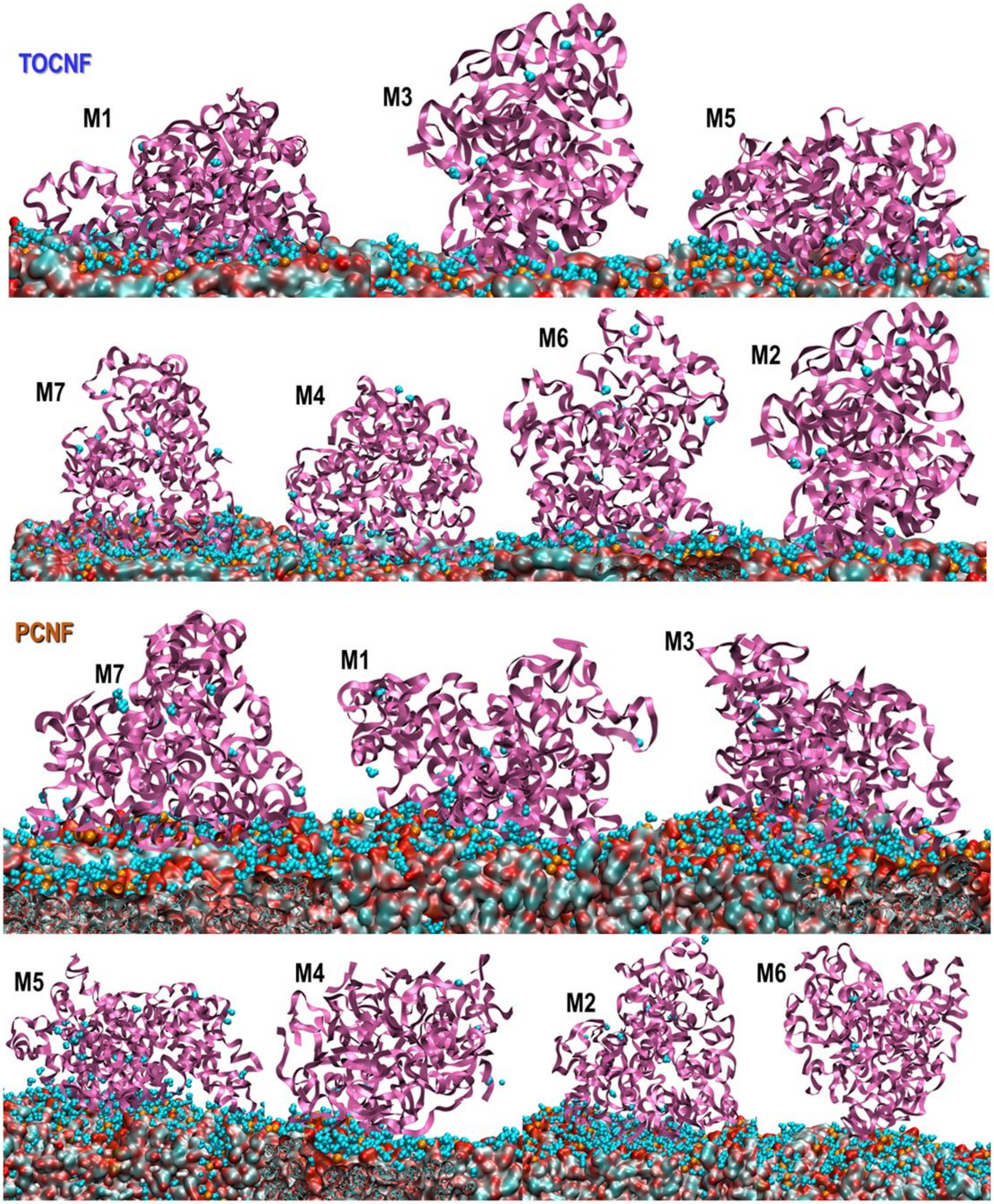

Figure S5. Possible adsorption modes of BSA on the cellulose functionalized interfaces (TOCNF and PCNF). The protein is rendered with a ribbon evidencing its secondary structure; water molecules (cyan) and counterions (orange) are rendered with vdW spheres, whereas the cellulose supports are displayed as solvent-accessible solid surfaces.

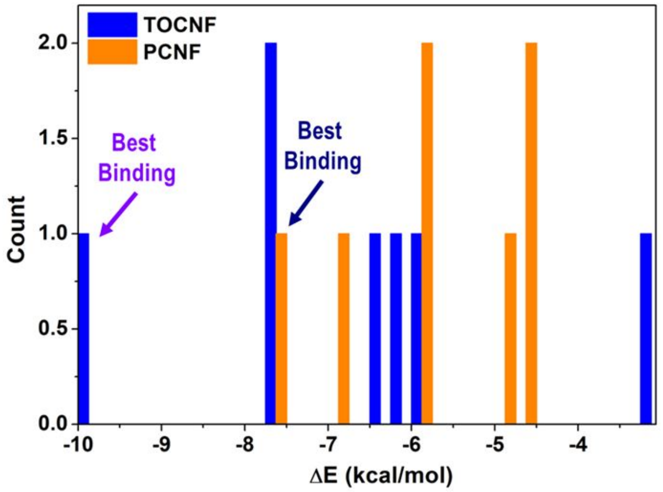

TOCNF	M1	M2	M3	M4	M5	M6	M7	mean	sigma	min.	max.
ARG	**4**	1	5	2	2	2	2	2.6	1.4	1	5
ASP	**16**	5	7	6	7	10	7	8.3	3.7	5	16
ASH	**0**	1	0	0	0	3	0	0.6	1.1	0	3
LYS	**5**	1	4	1	3	2	6	3.1	1.9	1	6
LYN	**11**	6	11	11	7	12	5	9.0	2.9	5	12
GLU	**10**	6	12	10	7	4	5	7.7	3.0	4	12
GLH	**4**	1	3	2	1	2	2	2.1	1.1	1	4
HIP	**4**	2	5	2	1	2	4	2.9	1.5	1	5

PCNF	M1	M2	M3	M4	M5	M6	M7	mean	sigma	min.	max.
ARG	3	2	2	2	2	1	**2**	2.0	0.6	1	3
ASP	9	6	6	6	5	2	**11**	6.4	2.9	2	11
ASH	0	3	0	0	0	1	**1**	0.7	1.1	0	3
LYS	3	3	3	0	8	5	**3**	3.6	2.4	0	8
LYN	9	10	8	9	3	3	**11**	7.6	3.2	3	11
GLU	8	6	9	1	10	5	**5**	6.3	3.0	1	10
GLH	3	2	3	1	3	2	**2**	2.3	0.8	1	3
HIP	3	3	3	1	5	2	**4**	3.0	1.3	1	5

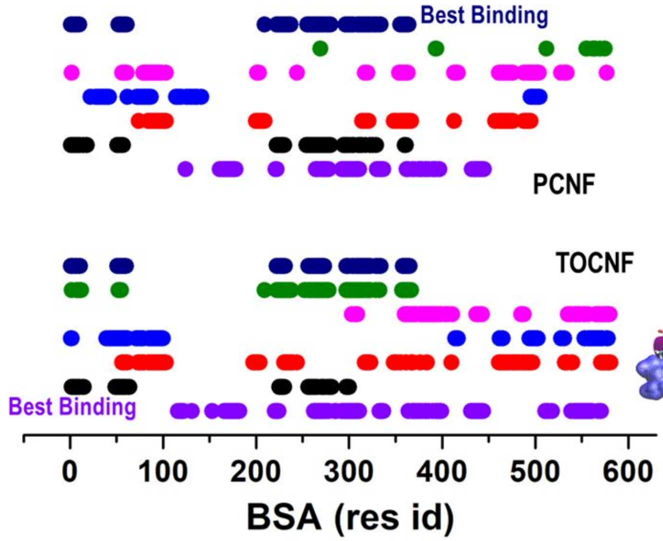

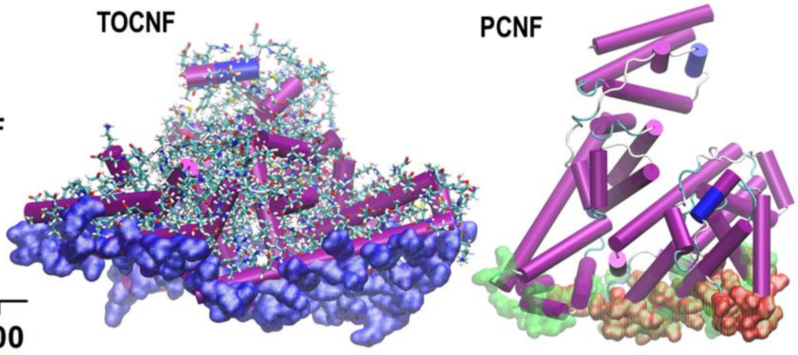

Figure S6. In the tables: number of basic (blue), acidic (red), and neutral (black) amino acids in the adsorbed portions of the protein. Istograms of the interaction energy per atom of the adsorbed regions of BSA on the two cellulose models. Comparison of the amino acid sequences adsorbed in the different models. The adsorbed areas in the best bindings models are highlighted on the original PDB of BSA (with intact secondary structure – magenta helices) by transparent solid vdW surfaces.

Table S3. Root mean square deviations of the Cα of the adsorbed BSA structures (Å) from the initial protein geometry.Solvent accessible surface areas (Å^2) with the corresponding numbers of residues for the isolated molecules (in the adsorbed geometry – final MD conformation) and the adsorbed portions. Interaction energies = Energy difference between the sum of the energy of the isolated BSA and cellulose support and the total energy of the complex divided by the total number of atoms. The best bindings are highlighted.

TOCNF	RMSD Cα	TOT sasa	ADS sasa	ADS sasa	TOT res	ADS res	ADS res	Int. En. x atom
	Å	Å^2	Å^2	%	#	#	%	kcal/mol
**M1**	**6.5**	**12726**	**3888**	**31**	**171**	**129**	**75**	**-9.1**
**M2**	10.5	10748	1519	14	142	51	36	-3.2
**M3**	9.5	11120	3165	28	149	113	76	-7.6
**M4**	16.3	12903	2630	20	172	80	46	-6.2
**M5**	7.1	12597	3077	24	172	83	48	-7.6
**M6**	6.2	12313	2581	21	161	80	50	-6.0
**M7**	6.4	13855	1972	14	187	71	38	-6.4

PCNF	RMSD Cα	TOT sasa	ADS sasa	ADS sasa	TOT res	ADS res	ADS res	Int. En. x atom
	Å	Å^2	Å^2	%	#	#	%	kcal/mol
**M1**	6.2	13812	2272	16	186	84	45	-6.8
**M2**	7.3	12475	1942	16	175	69	39	-4.6
**M3**	7.2	12677	1490	12	175	73	42	-5.9
**M4**	14.1	11048	1396	13	137	56	41	-4.8
**M5**	5.3	14947	2752	18	203	79	39	-5.9
**M6**	8.6	12054	331	3	158	11	7	-4.6
**M7**	**7.7**	**12465**	**2407**	**19**	**170**	**85**	**49**	**-7.7**

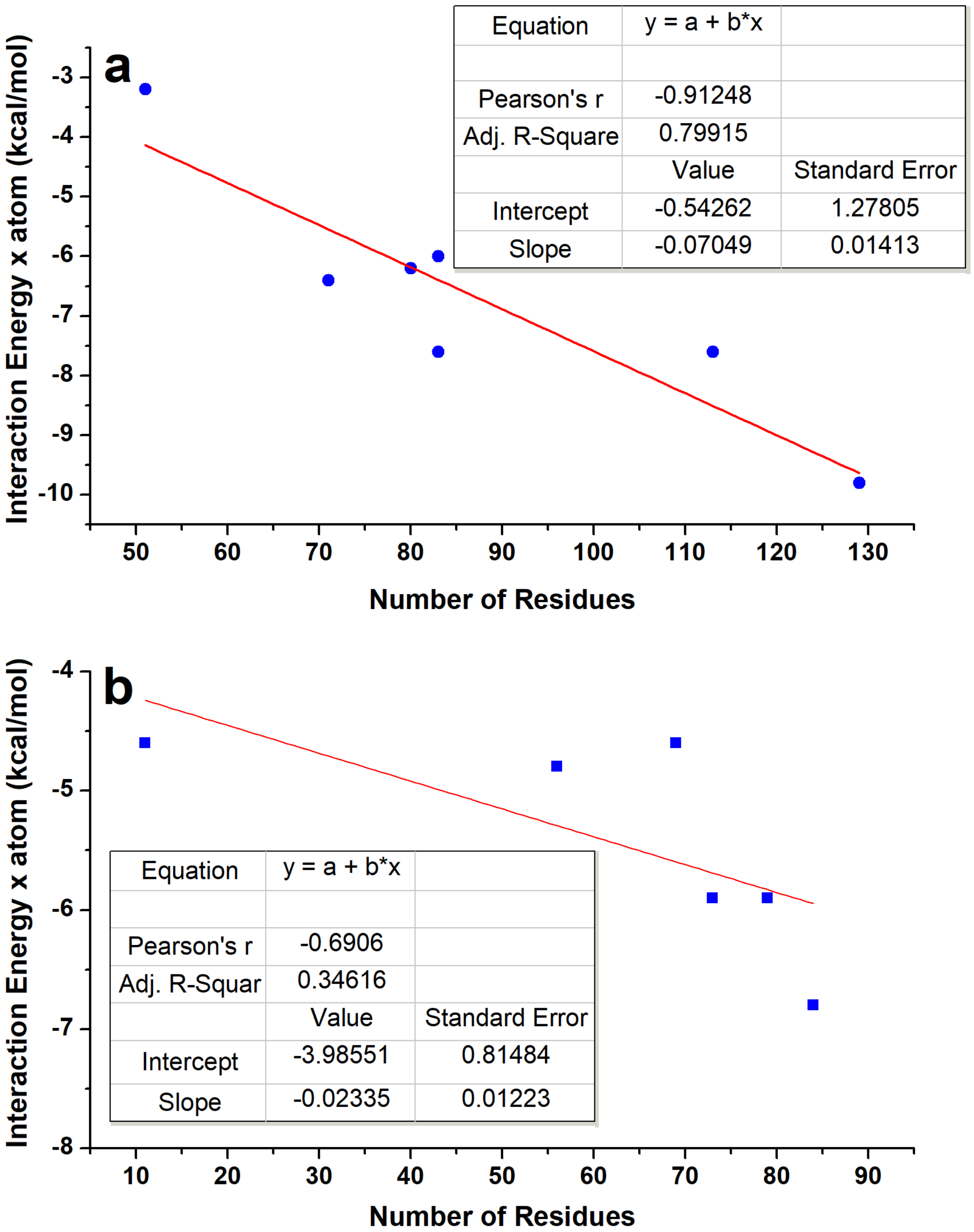

Figure S7.Interaction energy as a function of the number of adsorbed residues within 5.5 Å of the cellulose surface for the TOCNF (a) and PCNF (b) models.

Table S4. Disulfide bridges in the original PDB structure

Res. name	Res. #	Res. name	Res. #	S-S dist. (Å)
CYX	51	CYX	60	2.03
CYX	73	CYX	89	2.06
CYX	88	CYX	99	2.05
CYX	121	CYX	166	2.05
CYX	165	CYX	174	2.08
CYX	197	CYX	243	2.04
CYX	242	CYX	250	2.03
CYX	262	CYX	276	2.05
CYX	275	CYX	286	2.05
CYX	313	CYX	358	2.04
CYX	357	CYX	366	2.04
CYX	389	CYX	435	2.04
CYX	434	CYX	445	2.05
CYX	458	CYX	474	2.03
CYX	473	CYX	484	2.06
CYX	511	CYX	556	2.03
CYX	555	CYX	564	2.04

Table S5. Helical regions in the original PDB structure

Starting amino acid	Res. #	Ending Amino acid	Res. #	Residue length
SER	3	LEU	29	27
PRO	33	ASP	54	22
SER	63	LYS	74	12
SER	77	GLY	83	7
ASP	84	LYS	91	8
PRO	94	SER	102	9
ASP	116	ASP	127	12
ASP	127	HIS	143	17
TYR	147	CYS	166	20
ASP	170	PHE	220	51
GLU	224	HIS	244	21
ASP	246	ASN	264	19
GLN	265	ILE	268	4
GLU	274	LYS	278	5
PRO	279	GLU	289	11
PRO	301	ALA	307	7
ASP	311	ALA	319	9

Table S6. Amino acids sequence of the adsorbed portion of BSA (within 5.5 Å of the TOCNF surface). The helical regions are highlighted for the best interacting model (M1) (green filling).

TOCNF

**M1**	
ASP	116
PRO	117
ASN	118
THR	119
LEU	120
CYX	121
ASP	122
PHE	131
TYR	153
CYX	166
GLN	167
ALA	168
GLH	169
ASP	170
LYS	171
GLY	172
ALA	173
CYX	174
LEU	175
LEU	176
PRO	177
LYS	178
GLU	180
THR	181
MET	182
PHE	220
PRO	221
LYS	222
GLU	224
CYX	262
ASP	263
ASN	264
GLN	265
ASP	266
ILE	268
SER	269
SER	270
LYS	271
LEU	272
LYS	273
GLH	274
CYX	275
CYX	276
ASP	277
LYS	278
HIP	285
CYX	286
LYS	292
ASP	293
ALA	294
ILE	295
PRO	296
GLH	297
ASN	298
LEU	299
PRO	300
PRO	301
LEU	302
THR	303
ALA	304
ASH	305
ALA	307
GLU	308
ASP	309
ASP	311
ARG	333
ARG	334
HIP	335
PRO	336
GLU	337
HIP	364
SER	368
THR	369
VAL	370
PHE	371
ASP	372
LYS	375
HIP	376
ASP	379
GLN	382
ASN	383
LYS	386
ASP	390
GLH	393
LYS	394
LEU	395
GLY	396
GLU	397
TYR	398
GLY	399
GLY	431
THR	432
ARG	433
CYX	434
CYX	435
THR	436
LYS	437
PRO	438
GLU	439
SER	440
GLU	441
ARG	442
PRO	444
CYX	511
THR	512
LEU	513
PRO	514
ASP	515
LYS	518
GLU	539
LYS	542
THR	543
MET	545
GLU	546
ASN	547
VAL	549
ALA	550
VAL	552
ASP	553
LYS	554
CYX	555
CYX	556
ALA	557
ALA	558
ASP	559
ASP	560
LYS	561
GLU	568
LYS	571
**M2**	
LYS	2
SER	3
GLU	4
ILE	5
HIP	7
ARG	8
LYS	10
ASH	11
GLU	14
GLU	15
LYS	49
CYX	51
VAL	52
ALA	53
ASP	54
GLU	55
SER	56
HIP	57
ALA	58
GLY	59
GLU	61
LEU	64
PHE	225
VAL	226
GLU	227
VAL	228
THR	229
LYS	230
ASH	256
LEU	257
ALA	258
LYS	259
ILE	261
CYX	262
ASP	263
ASN	264
GLN	265
ASP	266
SER	270
LYS	271
LEU	272
GLH	274
ASP	277
LYS	278
PRO	279
LEU	280
LEU	281
GLH	297
ASN	298
LEU	299
PRO	300
**M3**	
SER	56
HIP	57
ALA	58
GLU	61
LYS	62
CYX	73
VAL	75
ALA	76
SER	77
LEU	78
ARG	79
GLH	80
ASP	84
MET	85
ALA	86
ASP	87
CYX	89
GLH	90
LYS	91
GLN	92
GLU	93
GLU	95
ARG	96
ASN	97
GLU	98
PHE	100
LEU	101
SER	102
HIP	103
CYX	197
ALA	198
SER	199
ILE	200
GLN	201
LYS	202
PHE	203
GLY	204
LYS	230
LEU	231
VAL	232
THR	233
ASP	234
THR	236
LYS	237
VAL	238
LYS	240
HIP	244
GLN	317
ALA	319
LYS	320
ASP	321
ALA	322
PHE	323
LYS	348
GLU	349
GLU	351
ALA	352
GLH	355
GLH	356
ASP	361
ASP	362
TYR	367
SER	368
THR	369
LEU	377
LEU	384
ARG	410
HIP	461
GLU	462
LYS	463
THR	464
PRO	465
VAL	466

SER	467
GLU	468
LYS	469
VAL	470
LYS	472
THR	475
GLU	476
SER	477
LEU	478
VAL	479
ASN	480
ARG	481
ARG	482
PRO	483
SER	486
ALA	487
LEU	488
THR	489
PRO	490
ASP	491
GLU	492
THR	493
TYR	494
VAL	495
PRO	496
LYS	497
HIP	532
LYS	533
LYS	535
GLU	539
GLN	540
LYS	571
VAL	573
VAL	574
THR	576
GLN	577
THR	578
ALA	579
LEU	580
ALA	581
**M4**	
HIP	1
LYS	2
LYS	39
ASN	42
GLH	43
THR	45
GLU	46
PHE	47
LYS	49
THR	50
CYX	51
VAL	52
ALA	53
ASP	54
SER	56
HIP	57
ALA	58
GLY	59
CYX	60
GLU	61
LYS	62
GLU	71
LEU	72
LYS	74
VAL	75
ALA	76
SER	77
LEU	78
ARG	79
MET	85
ALA	86
ASP	87
CYX	89
GLH	90
LYS	91
GLN	92
GLU	93
PRO	94
GLU	95
ARG	96
ASN	97
GLU	98
CYX	99
PHE	100
GLN	414
THR	417
GLU	462
PRO	465
TYR	494
VAL	495
PRO	496
LYS	497
ALA	498
PHE	499
ASP	500
LYS	502
LEU	503
GLU	528
LYS	531
ASP	553
LYS	554
ALA	557
ALA	558
ASP	559
ASP	560
LYS	561
GLU	562
ALA	563
CYX	564
PHE	565
ALA	566
VAL	567
GLU	568
GLY	569
PRO	570
LYS	571
LEU	572
VAL	573
GLN	577
THR	578
**M5**	
THR	303
ALA	307
GLU	308
ASP	309
LYS	360
ASP	361
ASP	362
PRO	363
ALA	365
CYX	366
TYR	367
SER	368
THR	369
VAL	370
PHE	371
ASP	372
LYS	373
HIP	376
LEU	377
ASP	379
GLU	380
GLN	382
ASN	383
LEU	384
ILE	385
LYS	386
GLN	387
ASN	388
ASP	390
GLN	391
PHE	392
GLH	393
LYS	394
LEU	395
GLY	396
TYR	398
GLY	399
ALA	403
LEU	404
ARG	407
LYS	411
LYS	437
PRO	438
GLU	439
SER	440
ARG	442
MET	443
PHE	485
SER	486
ALA	487
LEU	488
LYS	535
ALA	536
THR	537
GLU	538
GLU	539
LYS	542
THR	543
VAL	544
MET	545
GLU	546
ASN	547
VAL	549
VAL	552
ASP	553
LYS	554
ALA	557
PHE	565
ALA	566
VAL	567
GLU	568
GLY	569
PRO	570
LYS	571
LEU	572
VAL	573
VAL	574
SER	575
THR	576
GLN	577
THR	578
ALA	579
LEU	580
**M6**	
LYS	2
HIP	7
ARG	8
PHE	9
LYS	10
ASH	11
LEU	12
VAL	52
ALA	53
ASP	54
GLU	55
LYS	209
LYS	222
ALA	223
GLU	224
PHE	225
VAL	226
GLU	227
THR	229
LYS	230
LEU	231
VAL	232
THR	233
ASP	234
LEU	235
THR	236
LYS	237
VAL	238
ASP	252
ASP	253
ASH	256
LEU	257
LYS	259
TYR	260
ASP	263
ASN	264
GLN	265
ASP	266
THR	267
ILE	268
SER	269
SER	270
LYS	271
LEU	272
LYS	273
CYX	275
CYX	276
LYS	278
GLH	297
ASN	298
LEU	299
PRO	300
PRO	301
LEU	302
THR	303
ALA	304
ASH	305
PHE	306
ALA	307
GLU	308
ASP	309

LYS	310
ASP	311
VAL	312
CYX	313
LYS	314
ASN	315
GLH	318
ALA	319
LYS	320
ASP	321
PHE	323
TYR	329
ARG	333
CYX	357
CYX	358
ALA	359
LYS	360
ASP	361
ASP	362
PRO	363

HIP	364
TYR	367
**M7**	
HIP	1
LYS	2
SER	3
ALA	6
HIP	7
LYS	10
ASH	11
CYX	51
VAL	52
ALA	53
ASP	54
GLU	55
SER	56
HIP	57
ALA	58
CYX	60

LYS	222
GLU	224
PHE	225
VAL	226
GLU	227
VAL	228
THR	229
LYS	230
LEU	231
ASH	256
LEU	257
LYS	259
TYR	260
CYX	262
ASP	263
ASN	264
GLN	265
ASP	266
THR	267
SER	269

SER	270
LYS	271
LEU	272
LYS	273
GLH	297
ASN	298
PRO	300
THR	303
ALA	304
ASH	305
GLU	308
ASP	309
LYS	310
ASP	311
CYX	313
LYS	314
ASN	315
TYR	316
GLN	317
GLH	318

ALA	319
LYS	320
ALA	322
PHE	323
TYR	329
GLU	330
ARG	333
ARG	334
CYX	358
LYS	360
ASP	361
ASP	362
PRO	363
HIP	364
ALA	365

Table S7. Amino acids sequence of the adsorbed portion of BSA (within 5.5 Å of the PCNF surface). The helical regions are highlighted for the best interacting model (M7) (green filling).

PCNF

**M1**	
PHE	124
VAL	161
GLU	164
CYX	165
GLN	167
ALA	168
GLH	169
ASP	170
LYS	171
GLY	172
ALA	173
CYX	174
LEU	176
PRO	177
LYS	178
PRO	221
LYS	222
ASN	264
GLN	265
ASP	266
THR	267
SER	270
LYS	271
LEU	272
LYS	273
GLH	274
CYX	275
CYX	276
ASP	277
LYS	278
LYS	292
ASP	293
ALA	294
ILE	295
PRO	296
GLH	297
ASN	298
LEU	299
PRO	300
PRO	301
LEU	302
THR	303
ALA	304
PHE	306
ALA	307
GLU	308
ASP	309
LYS	310
ASP	311
GLU	330
TYR	331
ARG	333
ARG	334
HIP	335
PRO	336
GLU	337
ASP	362
HIP	364
SER	368
PHE	371
LYS	375
HIP	376
VAL	378
ASP	379
GLN	382
ASN	383
LYS	386
ASP	390
PHE	392
GLH	393
LYS	394
GLU	397
THR	432
CYX	435
THR	436
LYS	437
PRO	438
GLU	439
SER	440
GLU	441
ARG	442
MET	443
PRO	444
CYX	445
**M2**	
HIP	1
LYS	2
SER	3
GLU	4
ILE	5
ALA	6
HIP	7
ARG	8
PHE	9
LYS	10
ASH	11
GLU	14
GLU	15
LYS	18
CYX	51
VAL	52
ALA	53
ASP	54
GLU	55
HIP	57
LYS	222
GLU	224
PHE	225
VAL	226
GLU	227
THR	229
LYS	230
ARG	254
ALA	255
ASH	256
LEU	257
ALA	258
LYS	259
TYR	260
CYX	262
ASP	263
ASN	264
GLN	265
ASP	266
THR	267
ILE	268
SER	270
LYS	271
LEU	272
LYS	273
GLH	274
CYX	276
ASP	277
LYS	278
PRO	279
LEU	280
ALA	294
PRO	296
ASN	298
PRO	300
THR	303
ALA	304
ASH	305
LYS	310
ASP	311
VAL	312
CYX	313
LYS	314
GLH	318
PHE	323
SER	326
TYR	329
LYS	360
ASP	361
**M3**	
LYS	74
ASP	84
MET	85
ASP	87
CYX	88
GLH	90
LYS	91
GLN	92
GLU	93
PRO	94
GLU	95
ARG	96
GLU	98
CYX	99
PHE	100
LEU	101
SER	102
HIP	103
ILE	200
GLN	201
LYS	202
PHE	203
GLY	204
GLU	205
ARG	206
ALA	207
LYS	209
LYS	314
GLN	317
LYS	320
ASP	321
LYS	348
GLU	351
ALA	352
GLH	355
GLH	356
CYX	357
CYX	358
ALA	359
LYS	360
ASP	361
ASP	362
PRO	363
HIP	364
ALA	365
CYX	366
TYR	367
PRO	413
LEU	457
CYX	458
HIP	461
GLU	462
LYS	463
THR	464
PRO	465
VAL	466
SER	467
GLU	468
LYS	469
VAL	470
THR	471
LYS	472
CYX	474
THR	475
GLU	476
LEU	488
THR	489
PRO	490
ASP	491
GLU	492
THR	493
TYR	494
VAL	495
**M4**	
LEU	22
TYR	28
GLN	30
GLN	31
CYS	32
PRO	33
PHE	34
ASP	35
GLU	36
HIP	37
LYS	39
LEU	40
VAL	41
ASN	42
LYS	62
LEU	72

CYX	73
LYS	74
VAL	75
ALA	76
SER	77
LEU	78
ARG	79
GLH	80
THR	81
TYR	82
GLY	83
ASP	84
ALA	86
ASP	87
LYS	114
PRO	115
ASP	116
PRO	117
ASN	118
THR	119
LEU	120
LYS	125
ALA	126
ASP	127
LYS	130
PHE	131
LYS	134
TYR	135
TYR	137
ARG	141
VAL	495
PRO	496
LYS	497
ALA	498
PHE	499
ASP	500
LYS	502
LEU	503
PHE	504
THR	505
**M5**	
LYS	2
SER	56
HIP	57
ALA	58
GLY	59
GLU	61
ARG	79
GLH	80
THR	81
TYR	82
GLY	83
ASP	84
ALA	86

ASP	87
CYX	88
CYX	89
GLH	90
LYS	91
GLN	92
GLU	93
PRO	94
GLU	95
ARG	96
ASN	97
GLU	98
CYX	99
HIP	103
GLN	201
LYS	202
PHE	203
HIP	244
GLN	317
LYS	320
LEU	354
GLH	355
GLH	356
CYX	357
CYX	358
ALA	359
LYS	360
ASP	361
ASP	362
PRO	363
PRO	413
GLN	414
VAL	415
SER	416
THR	417
HIP	461
THR	464
PRO	465
VAL	466
SER	467
GLU	468
LYS	469
THR	471
LYS	472
THR	475
GLU	476
LEU	488
THR	489
GLU	492
THR	493
TYR	494
VAL	495
PRO	496
LYS	497

ALA	498
PHE	499
ASP	500
GLU	501
LYS	502
LEU	503
PHE	504
GLU	528
LYS	531
HIP	532
PRO	534
GLN	577
**M6**	
ASN	118
ALA	168
GLH	169
ASP	170
LYS	171
GLY	172
ALA	173
CYX	174
LEU	176
PRO	177
LYS	178
LYS	219
PHE	220
PRO	221
LYS	222
ASN	264
GLN	265
SER	270
LYS	271
LEU	272
LYS	273
GLH	274
VAL	290
GLU	291
LYS	292
ASP	293
ALA	294
ILE	295
PRO	296
GLH	297
ASN	298
LEU	299
PRO	300
PRO	301
ALA	304
ASH	305
ARG	334
HIP	335
GLU	337
PHE	371
HIP	376

GLN	382
ASN	383
THR	436
LYS	437
PRO	438
GLU	439
SER	440
GLU	441
**M7**	
HIP	1
LYS	2
SER	3
ALA	6
HIP	7
LYS	10
VAL	52
ALA	53
ASP	54
GLU	55
SER	56
HIP	57
ALA	58
GLY	59
CYX	60
GLU	61
LYS	209
LYS	222
GLU	224
PHE	225
VAL	226
GLU	227
VAL	228
THR	229
LYS	230
VAL	232
THR	233
ASP	234
THR	236
LYS	237
ALA	255
ASH	256
LEU	257
LYS	259
TYR	260
CYX	262
ASP	263
ASN	264
GLN	265
ASP	266
THR	267
ILE	268
SER	269
SER	270
LYS	271

LYS	273
CYX	276
ASP	277
LYS	278
PRO	279
LEU	280
PRO	296
ASN	298
LEU	299
PRO	300
PRO	301
THR	303
ALA	304
ASH	305
ASP	309
LYS	310
ASP	311
VAL	312
LYS	314
ASN	315
GLN	317
GLH	318
ALA	319
LYS	320
ASP	321
ALA	322
PHE	323
SER	326
PHE	327
TYR	329
GLU	330
ARG	333
ARG	334
GLH	355
GLH	356
CYX	358
LYS	360
ASP	361
ASP	362
HIP	364

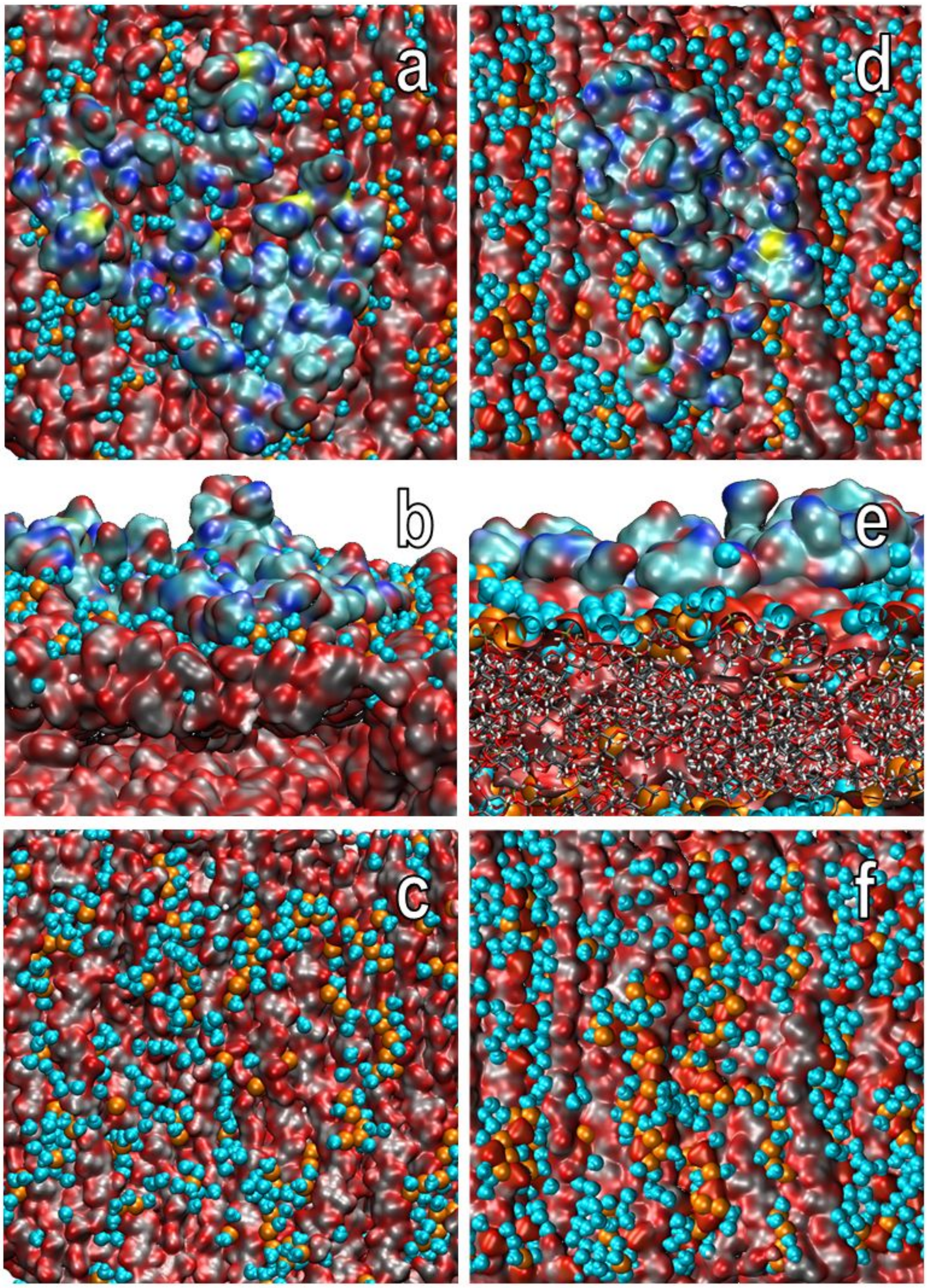

Figure S8. Top and side views of the adsorbed portions of BSA on TOCNF (a, b) and PCNF (d, e) with best interaction energy per atom (models M1 and M7, respectively). (c, f): the appearance of the cellulose surface under the adsorbed regions. Water molecules and Na+ ions are cyan and orange vdW spheres. The cellulose and BSA (aa within 5.5 A of the cellulose surface) are displayed as color-coded solvent-accessible surfaces. C gray, O red, N blue, S yellow, H white.
